# Emergence of Stable Synaptic Clusters on Dendrites Through Synaptic Rewiring

**DOI:** 10.3389/fncom.2020.00057

**Published:** 2020-08-06

**Authors:** Thomas Limbacher, Robert Legenstein

**Affiliations:** Institute of Theoretical Computer Science, Graz University of Technology, Graz, Austria

**Keywords:** dendrites, synaptic plasticity, structural plasticity, rewiring, synaptic clustering, catastrophic forgetting, neuroscience, spiking neural networks

## Abstract

The connectivity structure of neuronal networks in cortex is highly dynamic. This ongoing cortical rewiring is assumed to serve important functions for learning and memory. We analyze in this article a model for the self-organization of synaptic inputs onto dendritic branches of pyramidal cells. The model combines a generic stochastic rewiring principle with a simple synaptic plasticity rule that depends on local dendritic activity. In computer simulations, we find that this synaptic rewiring model leads to synaptic clustering, that is, temporally correlated inputs become locally clustered on dendritic branches. This empirical finding is backed up by a theoretical analysis which shows that rewiring in our model favors network configurations with synaptic clustering. We propose that synaptic clustering plays an important role in the organization of computation and memory in cortical circuits: we find that synaptic clustering through the proposed rewiring mechanism can serve as a mechanism to protect memories from subsequent modifications on a medium time scale. Rewiring of synaptic connections onto specific dendritic branches may thus counteract the general problem of catastrophic forgetting in neural networks.

## 1. Introduction

Long-term imaging studies of the living brain have revealed that the cortical connectivity structure is dynamic, with dendritic spines being added and deleted on the time scale of hours to days (Holtmaat et al., 2005; Stettler et al., 2006; Kasai et al., 2010; Loewenstein et al., 2011; Rumpel and Triesch, 2016). It has been proposed that this ongoing cortical rewiring serves important functions for learning and memory (Chklovskii et al., 2004; DeBello, 2008). According to this view, synaptic rewiring defines the connectivity structure of cortical circuits and interacts with synaptic plasticity of established synaptic connections.

Many theoretical studies of cortical rewiring have explored the implications of rewiring on the network level using point neuron models (see section 3). However, rewiring can also shape the connectivity structure on the sub-cellular level, defining the dendritic targets of synaptic connections onto pyramidal cells (PCs). As dendrites of cortical PCs exhibit various types of non-linear regenerative events, so-called dendritic spikes (Larkum et al., 2009), the specific placement of synaptic connections at the dendritic tree of a PC can strongly impact its computational function (Mel, 1994; Kastellakis et al., 2015; Bono et al., 2017). In particular, with regard to the organization of synapses on dendritic branches, a popular hypothesis with strong experimental support is the synaptic clustering hypothesis, which states that functionally related synapses tend to cluster on dendritic branches (Govindarajan et al., 2006; Kastellakis et al., 2015). A theoretical justification for rewiring of synapses on non-linear dendrites was provided by Poirazi and Mel (2001). There, it was shown that a supervised structural plasticity rule can optimize memory performance of a simple non-spiking model of a pyramidal cell with non-linear dendrites. Later, Legenstein and Maass (2011) showed that branch-specific synaptic plasticity—without synaptic rewiring—can self-organize non-linear computations in neurons with non-linear branches.

In this article, we study synaptic rewiring based on simple synaptic plasticity rules in a spiking neuron model for PCs with dendritic non-linearities. In computer simulations of the model, we find that rewiring leads to a clustering of temporally correlated inputs onto dendritic branches, thus supporting the synaptic clustering hypothesis. Using this model, we argue that synaptic clustering through rewiring could play an important functional role in learning processes. It was shown by Cichon and Gan (2015) that different motor learning tasks induce dendritic spikes in different dendritic branches of PCs in mouse motor cortex. A possible explanation for this finding is that synaptic inputs are clustered onto dendritic branches in a task-specific manner such that a dendritic branch receives predominantly synaptic inputs that are activated at a specific task. This interpretation is consistent with the synaptic clustering hypothesis. When this segregation was pharmacologically disrupted, the animals could still learn the current task, but performance on an earlier learned task degraded, indicating a role of clustering for retaining older memories. One may thus hypothesize that synaptic clustering provides a mechanism to shelter previous memories from being overwritten by novel plasticity events. Testing this hypothesis, we found in another series of computer simulations that synaptic clustering through our simple rewiring dynamics is able to shelter previous memories from subsequent modifications.

To model synaptic rewiring, we take advantage of the synaptic sampling theory (Kappel et al., 2015), which provides a general framework for the interaction of synaptic plasticity and rewiring. Synaptic plasticity in this framework has a strong stochastic component (Yasumatsu et al., 2008; Dvorkin and Ziv, 2016). This stochasticity naturally gives rise to a stochastic addition and deletion of synaptic connections, which can be shown to implement a stochastic search over connectivity structures. In our application of this framework, we combine rewiring with simple plasticity rules where plasticity events are triggered by local dendritic spikes (Golding et al., 2002; Lisman and Spruston, 2005; Gordon et al., 2006). While the branch-specific plasticity mechanism inevitably leads to the independent self-organization of each dendritic branch, we show that the addition of a spike-timing-dependent plasticity mechanism, as proposed by Legenstein and Maass (2011), can orchestrate this process across branches, leading to an increased capacity to store input patterns.

To probe conditions under which clustering emerges in our model, we tested the model in various situations. While synaptic clustering can occur under various input statistics, we observed no clustering when individual synaptic inputs were activated in an unreliable stochastic manner. Since these statistics are presumably more characteristic for early sensory areas, this may explain why some studies reported a lack of synaptic clustering (Jia et al., 2010). Finally, we theoretically analyzed the consequences of rewiring in our model. We found that the proposed dynamics approximates a stochastic search over connectivity structures that favors functionally clustered synaptic configurations.

## 2. Results

### 2.1. Synaptic Rewiring on Dendritic Branches

**Figure 1:**
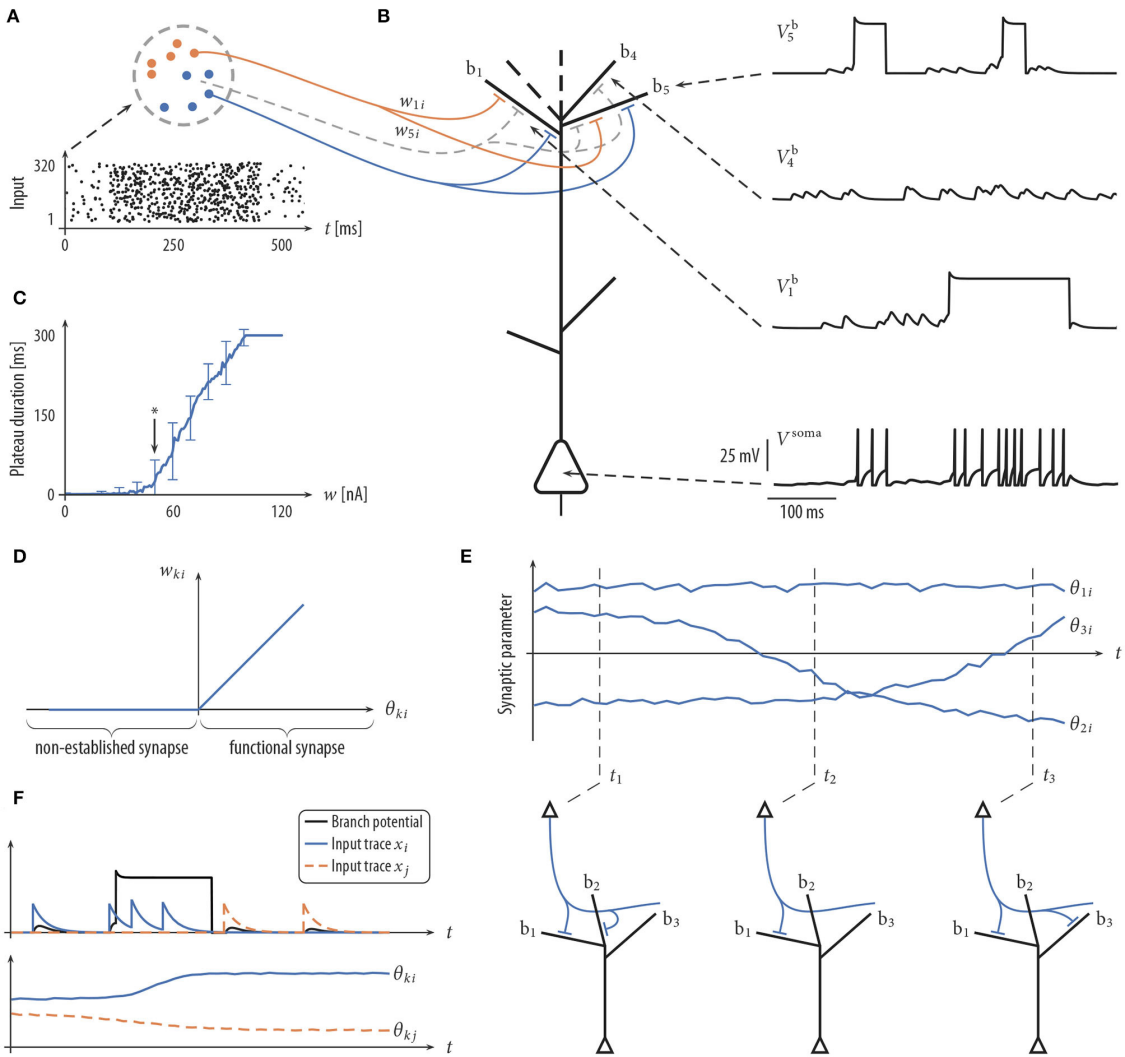
Schema of neuron model and plasticity/rewiring. **(A)** Input neurons (colored dots) are divided into different input assemblies (2 shown; color indicates assembly assignment). Bottom: spike raster of input neurons. Dots represent spike times. **(B)** Schematic drawing of neuron model (spatial structure of branches for illustrative purposes only) with dendritic membrane potentials $V_{i}^{\text{b}}$ (top right) and somatic membrane potential *V*^soma^ (bottom right). Branch b_5_ emits two dendritic spikes with a duration of about 50 ms. Branch b_1_ spikes once with a longer plateau phase. No synaptic cluster was established on branch b_4_. Therefore, this branch did not elicit dendritic spikes. Somatic spikes are indicated for illustrative purpose by vertical lines. **(C)** The duration of a plateau potential grows linearly as a function of the input intensity. The synaptic efficacy *w* of a single synaptic input was varied between 0 and 120 nA evenly spaced; a single strong synapse was used to mimic strong synchronous input. Shown is the mean and standard deviation of the resulting plateau durations over 100 independent trials. Arrow (at 50 nA) roughly marks the onset of this linear growth. Synaptic input exceeding this threshold has a fair chance of triggering a dendritic spike. **(D)** Mapping between the synaptic parameter θ_*ki*_ and the synaptic efficacy *w*_*ki*_ of synapse *i* onto branch *k*. Negative values of θ_*ki*_, corresponding to non-established synapses, are mapped to zero in the *w*_*ki*_-space. **(E)** Evolution of three synaptic parameters as a function of time *t* (top) and the corresponding wiring diagram at three points in time (bottom). At time *t*_1_ the values of parameters θ_1*i*_ and θ_2*i*_ are positive (indicating a functional synapse) while the value of parameter θ_3*i*_ is negative (indicating a non-established synapse). Parameter θ_2*i*_ crosses zero shortly before *t*_2_ (becoming non-established). **(F)** The plasticity process. Branch potential (black; sub-threshold potentials and a dendritic spike), the somatic spike traces of input neurons *i* and *j* (top), and the evolution of synaptic parameters θ_*ki*_ and θ_*kj*_ (bottom). Input neuron *i* is active shortly before and during the dendritic spike. The synaptic parameter θ_*ki*_ of this connection is therefore increasing. θ_*kj*_ decreases, since neuron *j* is not active during the plateau potential.

Dendritic structures of pyramidal cells (PCs) can be divided into integrative compartments (Losonczy and Magee, 2006; Major et al., 2008; Larkum et al., 2009; Branco et al., 2010). The compartmental model for PCs adopted in this article is illustrated in Figures 1A,B. The axon of a presynaptic neuron *i* can contact various dendritic branches *k* of the postsynaptic PC, establishing a synaptic connection *ki* with synaptic efficacy *w*_*ki*_. Presynaptic spikes at such a synapse *ki* give rise to alpha-shaped currents of amplitude *w*_*ki*_ at the branch. Each dendritic branch acts as a leaky integrator that temporally and spatially sums synaptic input currents from its incoming synapses. Note that we do not consider any specific spatial structure of the branches. The structures shown in the figures are provided for illustration purposes only.

Besides this passive response to synaptic inputs, dendrites of PCs show a variety of non-linear responses. In particular, we model local N-methyl-D-aspartate (NMDA) spikes (Antic et al., 2010) that include a brief sodium spikelet followed by a plateau potential that lasts up to a few hundreds of milliseconds, see Figure 1B [amplitude values of the plateau potentials are taken from Antic et al. (2010), see Figure 1 there]. NMDA spikes are elicited stochastically at dendritic branches based on the local membrane potential (branch potential). The duration of the spike grows linearly with stimulus intensity (Figure 1C), whereas the amplitude exhibits an all-or-none highly non-linear behavior (Antic et al., 2010). Currents from dendritic compartments to the soma are driven by the difference between the branch potential and the membrane potential of the soma. The somatic compartment is again a leaky integrator that sums currents from all dendrites (with a certain degree of amplitude attenuation, see Equation 17). Somatic action potentials are elicited stochastically with an instantaneous firing rate that depends exponentially on the somatic membrane potential. See Jolivet et al. (2006) for a fit of this model of action potential generation to layer V pyramidal neurons in rat somatosensory cortex. A more detailed description of the neuron model is given in section 4.

The connectivity structure in the cortex is not static, rather synaptic connections are rewired on a time scale of hours to days (Holtmaat et al., 2005; Stettler et al., 2006; Minerbi et al., 2009; Yang et al., 2009; Ziv and Ahissar, 2009; Kasai et al., 2010; Loewenstein et al., 2011; Rumpel and Triesch, 2016; Chambers and Rumpel, 2017). To investigate the consequences of rewiring processes for information processing in PCs, we adopted the synaptic sampling framework introduced by Kappel et al. (2015, 2018). Each compartment has a set of potential synaptic connections that could be realized by some parameter setting. However, at each point in time, only a subset of these connections is realized by functional synapses. More precisely, we maintain one parameter θ_*ki*_ for each potential synapse *ki* from input neuron *i* to branch *k* of the neuron. This parameter encodes (a) through its sign whether the synapse is functional and (b) the synaptic efficacy *w*_*ki*_ of the synapse if it is functional. More precisely, the synaptic weight *w*_*ki*_ is *c*_θ_θ_*ki*_ for a functional synapse (θ_*ki*_ > 0) and 0 otherwise (θ_*ki*_ ≤ 0). Hence, the mapping from the parameter θ_*ki*_ to the synaptic weight is given by *w*_*ki*_ = *c*_θ_max{0, θ_*ki*_} where *c*_θ_ = 1 nA is the slope of this mapping for θ_*ki*_ > 0 (Figure 1D).

Recent experimental results show that synaptic modifications contain a strong autonomous component (Dvorkin and Ziv, 2016). In accordance with previous modeling approaches (Loewenstein et al., 2011), this component is modeled in the synaptic sampling framework as a stochastic process. In general, the stochastic synaptic dynamics is given in this model as a stochastic differential equation (SDE) of the form

(1) $$\begin{array}{l} d\theta_{ki}\left(t\right)=\eta \left(f_{ki}^{S}\left(t\right)+f_{ki}^{L}\left(t\right)\right)dt+\sqrt{2\eta T}dW_{ki}, \end{array}$$

where η > 0 is a small learning rate (for parameter values, see Table 2). This SDE can be read as follows: the change of the synaptic parameter θ_*ki*_ is the sum of three terms. The last term models the synapse-autonomous component that does not depend on any synaptic, presynaptic, or postsynaptic variables. Instead, it is given by the increments of a standard Wiener process $W_{ki}$, thus implementing random walk behavior. The temperature *T* is a constant that scales the strength of this stochastic component. The term in the brackets defines the deterministic part of the dynamics. For conceptual clarity, it is divided here into two components. The first term $f_{ki}^{S}$ describes changes that are not directly relevant for the functional goal of the plasticity process but rather used to enforce structural constraints on the connectivity (for example, bound the number of connections that can be established onto one branch). The functional goal of the plasticity process is captured by the second term $f_{ki}^{L}$, see below for an example. The deterministic terms will be chosen such that they vanish for θ_*ki*_(*t*) ≤ 0. Hence, for non-established synapses, only the stochastic term remains. The random walk may at some point cross zero again, leading to the creation of a synaptic connection which may be strengthened or weakened depending on its functional relevance (Figure 1E). The model thus integrates synaptic dynamics with rewiring in one unifying framework. Note that the creation of new synapses solely depends on the Wiener process. New synapses can also directly be sampled from the set of non-established potential synapses, which however loses some of the nice mathematical properties of the model, see Bellec et al. (2018) for details.

Since in cortical PCs, the number of connections to a branch is bounded, we use for the structural component $f_{ki}^{S}$ of the plasticity dynamics (1) a term that enforces a soft upper bound on the number of synaptic connections onto each branch. This term depresses weak synapses of a branch if the number of connections to it is close to or exceeds a predefined constant *N*_syn_ (set to 20 in our simulations), and it vanishes if the number of connections is well below *N*_syn_.

According to the Hebbian theory of synaptic plasticity, synaptic connections are created and/or strengthened when the presynaptic neuron contributes to activity of the postsynaptic neuron (Hebb, 1949). More recent experiments, however, showed that postsynaptic somatic spiking is neither necessary nor sufficient to induce long-term potentiation (LTP) in pyramidal cells in various brain structures, both, at distal basal dendrites and at the apical tuft (Golding et al., 2002; Lisman and Spruston, 2005; Gordon et al., 2006; Gambino et al., 2014; Cichon and Gan, 2015; Brandalise et al., 2016). These studies indicate that local dendritic NMDA-dependent regenerative processes are necessary as the postsynaptic signal to trigger long-term potentiation (LTP). An important implication of this finding is that the learning signal is at least partially local to dendritic branches, leading to a synaptic organization that depends on local dendritic integration. To model such LTP within the stochastic rewiring framework, we include a term *x*_*i*_(*t*)Γ_*k*_(*t*) in the functional term $f_{ki}^{L}$ of the plasticity dynamics (1). Here, *x*_*i*_(*t*) is a trace of the spike train from presynaptic neuron *i*, and Γ_*k*_(*t*) = 1 indicates the presence of an NMDA plateau potential at time *t* (Γ_*k*_(*t*) = 0 otherwise). Hence, coincidence of presynaptic activity with a dendritic plateau potential will induce LTP (see Figure 1F, blue trace). While the dependence for LTP on dendritic spikes is well-established in the literature, less is known about long-term depression (LTD) in this regard. Interestingly, while Golding et al. (2002) observed LTP when pairing high-frequency stimulation of synaptic inputs with dendritic spikes in a theta-burst pairing protocol, Holthoff et al. (2004) reported long-term depression (LTD) with a single presynaptic stimulation leading to a dendritic spike, indicating that low-frequency pairing may lead to LTD, consistent with the classical findings that weak synaptic stimulation leads to LTD (Dudek and Bear, 1995) under the assumption that dendritic spikes are elicited in the postsynaptic neuron occasionally. We therefore included a term in our plasticity model that weakens a synapse if its activity is below a certain level during a plateau potential. Together, the functional component $f_{ki}^{L}$ of the plasticity dynamics (1) was given by

(2) $$\begin{array}{l} f_{ki}^{L}\\ =\{\begin{array}{ll} c_{L}\Gamma_{k}(t)(x_{i}(t) & \text{if }\theta_{ki}(t)>0\text{ (functional connection)}\\ -\gamma (1-x_{i}(t)), & \;\\ 0, & \text{if }\theta_{ki}(t)\leq 0\text{ (non-established connection)}, \end{array} \end{array}$$

with γ > 0 being a constant that determines the threshold activity that switches from LTD to LTP. Note that changes occur only during the presence of a plateau potential [Γ_*k*_(*t*) = 1]. The rule can be described qualitatively as follows: if there is a dendritic spike at branch *k*, potentiate active synapses and depress inactive synapses to this branch *k*. Weak synapses will ultimately retract, and non-established potential synapses will be established at random times according to the stochastic process. We show in the section “Analysis of stochastic rewiring dynamics” that this dynamic corresponds to a distribution $p_{L}$ that defines functionally preferred parameter settings that lead to synaptic clustering.

### 2.2. Synaptic Clustering Through Rewiring

**Figure 2:**
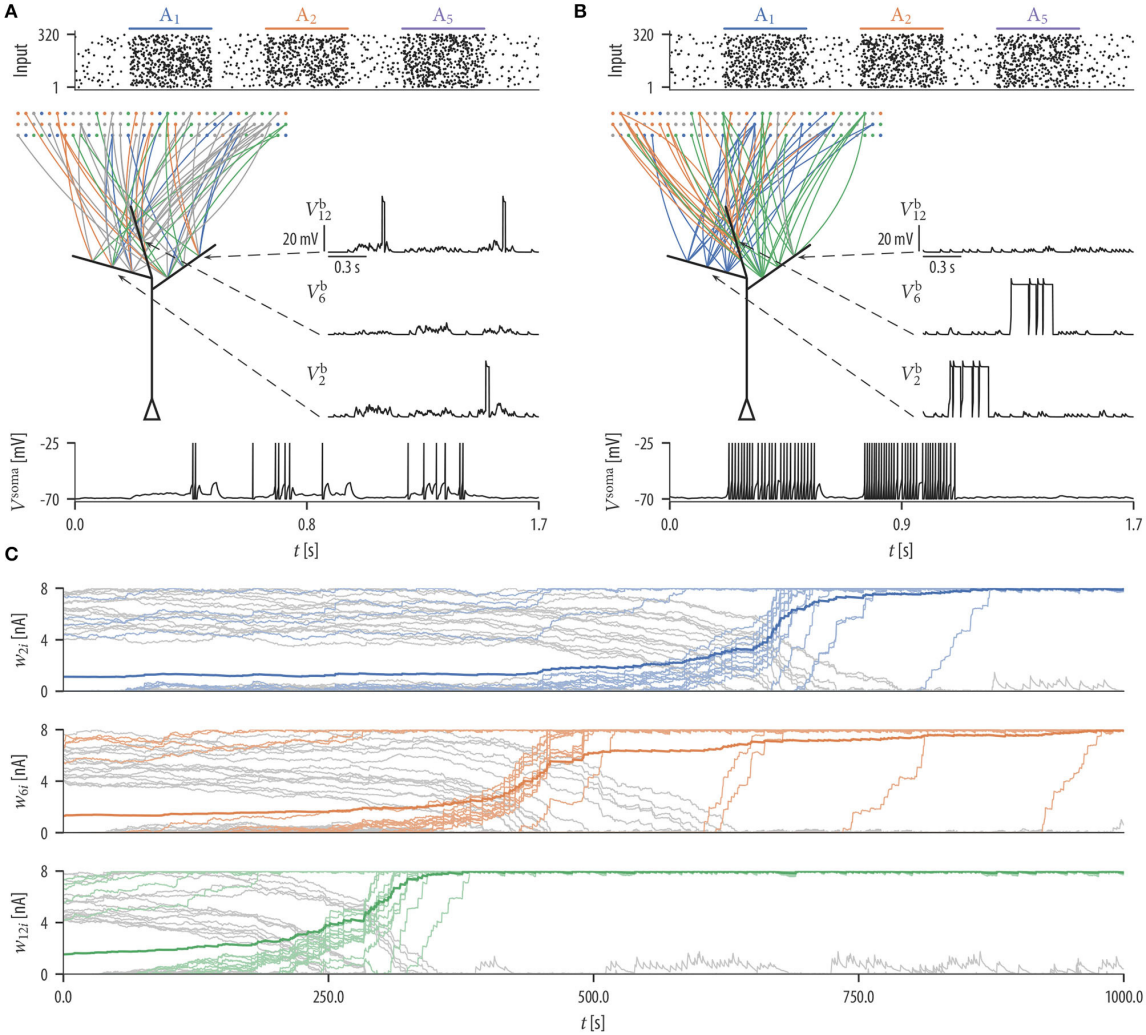
Synaptic rewiring leads to synaptic clustering at dendritic branches. **(A)** Spike rasters of input neurons (top), initial wiring diagram of three selected branches (middle left), dendritic membrane potentials $V_{i}^{\text{b}}$ of these branches (middle right), and the somatic membrane potential *V*^soma^ (bottom) for the successive activation of assemblies A_1_, A_2_, and A_5_. Color of graph edges indicate to which of the assemblies (A_1_, A_2_, and A_3_; pattern presentation of assembly A_3_ is not shown) a connection was established. Connections in gray are connections to one of the other assemblies. At this initial configuration, the input neurons were connected to branches randomly. Brief dendritic spikes appeared occasionally (middle right) and the neuronal firing rate was low (bottom). **(B)** After 17 min of rewiring dynamics, inputs to each branch were originating predominantly from a specific input assembly A_*i*_ (middle left). Hence, temporally correlated branch input triggered long lasting dendritic plateau potentials (middle right). In this simulation, none of the branches received synaptic clusters from assembly A_5_. As a consequence, the neuron elevated its firing rate during pattern presentations of input assemblies A_1_ and A_2_, but rarely spiked during the pattern presentation of assembly A_5_ (bottom). **(C)** Evolution of the synaptic weights *w*_*ki*_ of branch b_2_ (top), branch b_6_ (middle), and branch b_12_ (bottom). Shown is the mean synaptic weight from three selected assembly (A_1_, A_2_, and A_3_) which clustered on the branch (saturated color), weights of individual synapses from that assembly (desaturated color), and weights of synapses from other assemblies (gray). Colors correspond to colors in **(A,B)**. Synaptic connections are reorganized during the rewiring process, i.e., synapses with correlated activity are established on the branch while synapses of other assemblies retract over time. Occasional small gray bumps close to 0 in the second half of the simulation originate from synapses from other assemblies that are reconnected, but they quickly retract due to their irrelevance for activating the branch.

We first investigated the patterns of synaptic connections that emerge when synaptic rewiring is driven by the simple dendritic spike-dependent plasticity mechanism (2). We simulated a neuron with 12 independent dendritic compartments that received input from 320 input neurons. Input neurons were divided into 8 disjoint assemblies of 40 neurons each, producing Poisson spike trains at a background rate of 1 Hz. Every 500 ms, one assembly was chosen randomly and activated for 300 ms. These neurons elevated their firing rate to 35 Hz, leading to a specific input firing pattern (Poisson spike trains were randomly generated at every pattern presentation), see Figure 2A (top).

Input neurons were initially connected to dendritic branches randomly such that 20 synapses were established on each branch (Figure 2A middle). Synaptic parameters of these connections were independently drawn from a uniform distribution over the interval [4, 8). A soft upper bound of 20 synapses per branch was enforced through the structural component $f_{ki}^{S}$ of the synaptic sampling update (1). At this initial configuration, brief dendritic plateau potentials appeared occasionally and the neuronal firing rate was low (≈ 6.19 Hz). Dendritic plateau potentials were elicited preferentially during presynaptic assembly activations, but they were not specific to any of the input assemblies. We observed a gradual assembly-specific clustering of synapses onto individual branches during the simulation. After 17 min of assembly activations, each branch usually hosted a synaptic cluster from one of the input assemblies, receiving approximately 20 synapses from that assembly with the weight close to the maximum, while other assemblies were either not connected or had weights close to zero, see Figure 2B. Many synapses that were functional after rewiring were not connected to the branches initially but were established during the rewiring process (Figure 2C). In order to quantify the behavior of the model, we performed 25 simulations with different randomly chosen initial conditions and different random assembly activations. We say that assembly synapses cluster on a branch if the branch receives at least 10 synapses from one assembly with a total weight of at least 50 nA. The value of 50 nA was chosen since synaptic input exceeding this threshold has a fair chance of triggering a dendritic spike (see arrow in Figure 1C). In our 25 independent simulations, from the 8 input assemblies, between 5 and 8 were represented on dendritic branches with a mean of 6.36 and a standard deviation (SD) of 0.84. In all simulations, most of the dendritic branches had a synaptic cluster from one assembly, and some assemblies were clustered on several branches.

Since inputs to each branch were preferentially originating from a specific input assembly after rewiring, branch inputs were temporally correlated. This collective activity was sufficient to trigger long-lasting dendritic plateau potentials and somatic spikes (Figure 2B; voltage traces at active assemblies *A*_1_ and *A*_2_). In total, three branches received synaptic clusters from assembly A_2_ and two branches from assembly A_1_. This was reflected in the firing rate of the neuron during the activation of these assemblies. The firing rate of the neuron was highest (≈ 63.5 Hz) during the pattern presentation of assembly A_2_ and lowest (≈ 54.2 Hz) during the activation of assembly A_1_. To test the response of the neuron to assemblies that were not clustered, we activated assembly A_5_ that was active during the rewiring process but did not evolve a synaptic cluster on any branch. Branches did not elicit spikes during the pattern presentation of this assembly and the neuronal firing rate was low (≈ 0.97 Hz; Figure 2B; voltage traces at active assembly *A*_5_).

We hypothesized that dendritic plateau potentials are crucial for assembly-specific clustering of synapses in our model. To verify this we conducted simulations of an altered model with linear dendritic integration (i.e., the same model but without dendritic spikes). We did not observe clustering of functionally related inputs onto dendrites in this altered model on a large range of relevant parameters (see section 1.1 in the Supplementary Material for details), indicating that dendritic plateau potentials are indeed necessary for assembly-specific clustering of synapses onto individual dendrites.

In summary, these results show that synaptic rewiring dynamics can give rise to (a) clustering of functionally related inputs onto dendritic compartments and (b) segregation of different assemblies onto different branches. This leads to segregated assembly-specific activation of dendritic branches. Note that this plasticity-driven self-organization of network connectivity is independent of postsynaptic somatic spikes.

### 2.3. STDP Increases the Capacity of Neurons to Store Assembly Patterns

With the plasticity dynamics considered above, since plasticity depends solely on synaptic inputs and local dendritic potentials, all branches are adapted independently without taking the activity of other branches into account. This implies that synaptic patterns at different branches can become correlated in the sense that projections from one assembly cluster on two or more branches of the neuron. Such adaptation may in general waste dendritic resources such that all branches become occupied by a subset of input assemblies while other assemblies are neglected. This effect would become even more pronounced if assemblies were not activated in an intermixed manner but sequentially. In this case, early assemblies would occupy all branches, leaving no representational space for later ones.

Previous work has shown that dendritic synaptic patterns can be decorrelated by a simple additional spike-timing-dependent plasticity (STDP) mechanism where each somatic spike induces a small amount of depression in all synapses with a recent presynaptic spike (Legenstein and Maass, 2011). This rule is consistent with the experimental finding that in distal dendrites, a pre-before-post spike pair leads to depression (Kampa et al., 2007). The STDP update indirectly introduces competition between dendritic branches such that branches compete for becoming activated for each input pattern. Briefly, consider an input pattern for which one branch (or a few branches) has evolved a strong synaptic cluster. Whenever this input is presented, the branch becomes active, it produces dendritic plateau potentials which in turn will lead to somatic spikes. For branches that did not yet evolve a strong cluster for that input pattern, these somatic spikes then depress via STDP active synapses and thus prevent the emergence of a cluster for this pattern. On the other hand, the branch with the strong cluster and plateau potentials is protected in the sense that potentiation from the dendritic-spike-dependent plasticity rule overcompensates the depression. This branch (or a few branches with a strong cluster) has thus won the competition for becoming activated by this input pattern.

**Figure 3:**
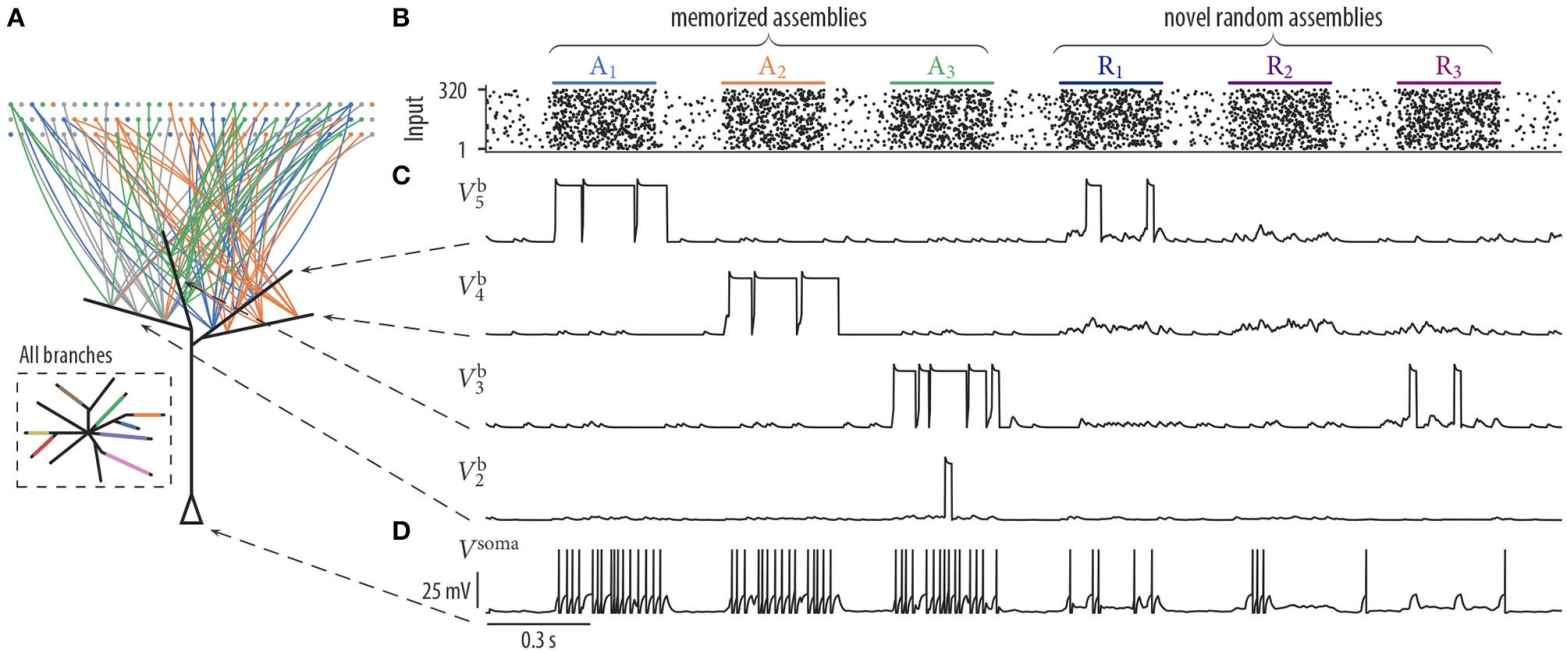
Synaptic rewiring with spike-timing-dependent depression and neuron response to stored and novel input patterns. **(A)** Schematic neuron drawing wiring diagram of four selected branches after 17 min of rewiring dynamics (as in Figure 2B). Branches b_3_, b_4_, and b_5_ received synaptic inputs predominantly from assemblies A_3_, A_2_, and A_1_, respectively. Branch b_2_ remained without a synaptic cluster from any assembly. Inset: Schematic drawing of all branches of the neuron. Branch color indicates assembly origin of a synaptic cluster. All assemblies were represented on the branches and each assembly established only a single cluster. Four branches remained without a synaptic cluster from any assembly. **(B–D)** Pattern-specific neuron responses after rewiring. **(B)** Input neurons spike raster. Assemblies *A*_1_, …, *A*_3_ were also activated during the previous rewiring period. The last three firing patterns were generated by novel random assemblies R_*i*_. **(C)** Dendritic membrane potentials $V_{i}^{\text{b}}$ of four branches during the presentation of three test patterns of assemblies A_*i*_ and R_*i*_. Dendritic plateau potentials were elicited preferentially during the activation of memorized assemblies A_*i*_ and appeared only occasionally at novel random assemblies R_*i*_. **(D)** Somatic membrane potential *V*^soma^ during the presentation of input patterns of memorized assemblies (A_1_, A_2_, and A_3_) and for patterns of novel random assemblies (R_1_, R_2_, and R_3_). The neuron responded with a high firing rate (≈ 27.6 Hz) to patterns of memorized assemblies and with a low firing rate (≈ 11.3 Hz) during the activation of novel random assemblies.

To test the effect of this mechanism on synaptic rewiring, we performed 25 simulations as described above but with STDP added to the parameter dynamics (see section 4 for a definition of the STDP update). We found that the average number of represented assemblies increased from 6.36 ± 0.84 to 7.40 ± 0.57 (mean ± SD). This is a significant increase of the number of assemblies that are stored on the dendrites [*t*_(24)_ = 5.01, *p* < 0.0001, unpaired *t*-test]. A schematic drawing of the simulated neuron and the wiring diagram after 17 min of rewiring dynamics of four selected branches of one of the conducted trials is shown in Figure 3A. Each assembly in this simulation evolved a synaptic cluster onto exactly one dendritic branch. Four branches did not receive a synaptic cluster from any of the assemblies (inset Figure 3A). Since these branches did not specialize to a specific input assembly, they could, in principle, adapt later to respond to novel input assemblies. To test whether these results are sensitive to the specific values of parameters in our rewiring model, we performed a sensitivity analysis. This analysis showed that the number of synaptic clusters is quite robust to parameter variations (see section 1.2 in the Supplementary Material). We also tested effects of the pattern duration and the delay between patterns. We found that our rewiring mechanism is, up to some point, quite robust to variations in the pattern duration and the delay between patterns (see section 1.3 in the Supplementary Material).

We next asked whether the neuron was able to differentiate after the rewiring process between stored and novel input patterns. We presented three firing patterns of memorized assemblies A_1_, A_2_, A_3_ and three firing patterns of novel random assemblies R_1_, R_2_, R_3_ to the neuron (Figure 3B). Each novel assembly was created by randomly choosing 40 distinct neurons from all 320 input neurons. Hence, assemblies R_1_ to R_3_ were not necessarily disjoint and they shared a significant amount of neurons with assemblies A_1_ to A_8_. The neuron responded with a high firing rate during the activation of memorized assemblies but spiked only occasionally for patterns of novel random assemblies (Figure 3D). Branches that did not receive a synaptic cluster from any of the assemblies still maintained rather strong individual synapses originating from multiple assemblies, but since their collective activity was not correlated; it was not sufficient to trigger long lasting dendritic plateau potentials in these branches (Figure 3C, bottom trace $V_{2}^{\text{b}}$).

In summary, STDP increases the capacity of the neuron in terms of the number of assemblies that are stored on dendrites. If there are more branches than assemblies in the input, some branches remain assembly-unspecific and are therefore available to store novel assemblies. Unless otherwise stated, all of the following simulations presented below are conducted with the STDP update.

### 2.4. Rewiring Protects Stored Information

One effect of the synaptic clustering observed in our simulations was that activation of different input assemblies led to dendritic spikes in different dendritic branches, see Figures 2A,B, 3B. Similarly, Cichon and Gan (2015) observed task-specific segregation of dendritic activity in tuft branches of layer V pyramidal neurons in mouse motor cortex. More specifically, they observed that different motor learning tasks induce dendritic spikes on different branches. The processing of different behaviors is therefore separated onto different dendritic branches. Importantly, when this segregation was pharmacologically disrupted, the animals could still learn the current task, but performance on an earlier learned task degraded, indicating a role of this clustering for retaining older memories. The authors thus argued that this segregation may provide a mechanism to shelter previous memories from being overwritten by novel plasticity events.

**Figure 4:**
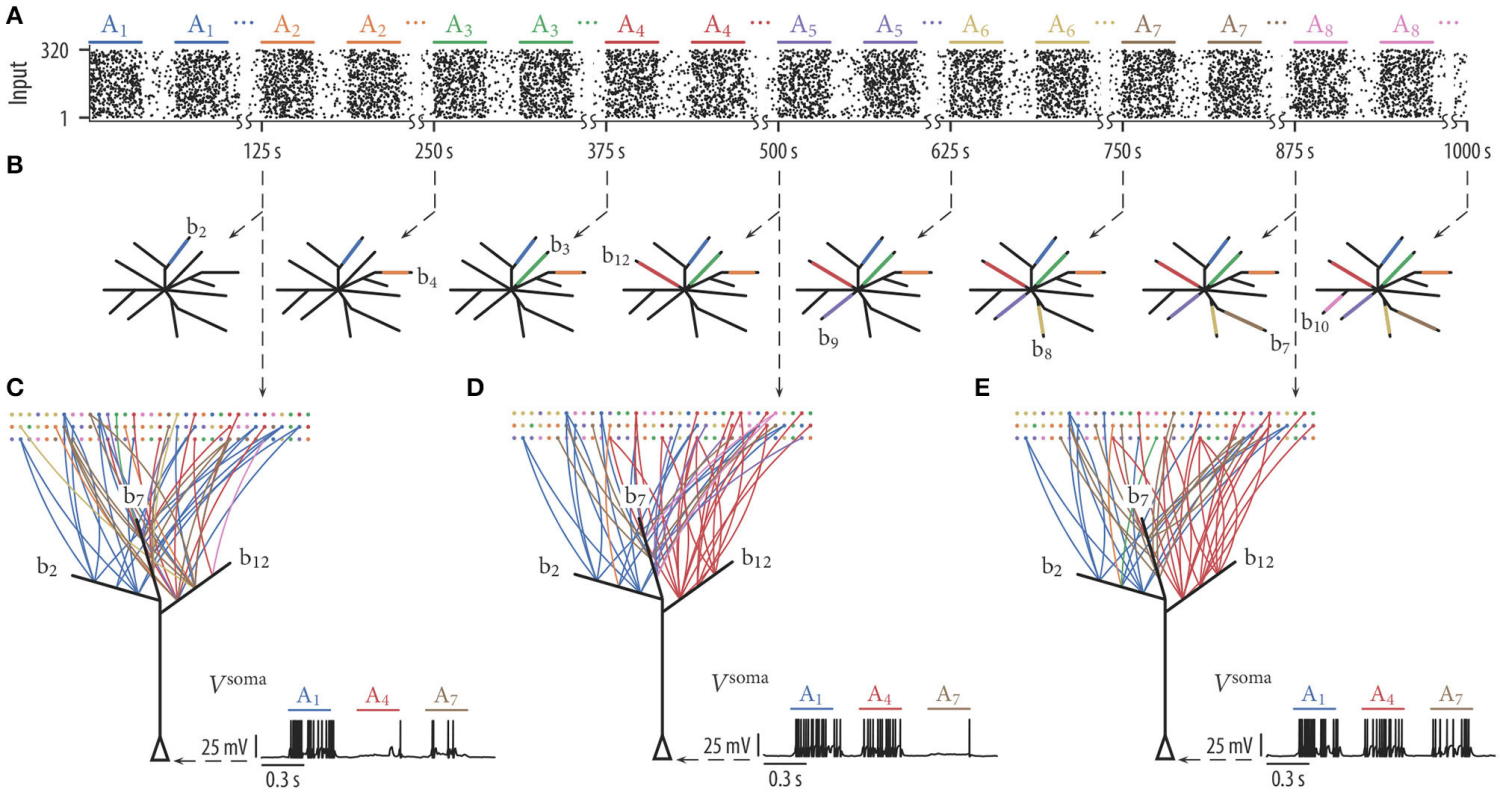
Dendritic-spike-dependent rewiring protects stored information by segregating functionally unrelated inputs onto different dendritic branches. When input assemblies were activated sequentially, novel assemblies clustered on free branches while previously established clusters were retained. **(A)** Input assemblies A_*i*_ activated in a sequential manner. We first activated exclusively assembly A_1_, then assembly A_2_, then A_3_, etc. **(B)** Schematic drawing of the 12 simulated branches at 8 different time points (branch color indicates assembly origin of synaptic cluster at the branch). Branches were recruited sequentially. **(C–E)** Network connectivity graph and somatic membrane potential *V*^soma^ of three selected branches (b_2_, b_7_, and b_12_) at three different time points. Graph edge color indicates to which of the input assemblies a connection is established. The somatic potential shows the response of the neuron to one test pattern of assembly A_1_, assembly A_4_, and assembly A_7_. The neuron successively reorganized its synaptic connections such that it responds to any of the patterns that have been presented previously.

To test whether simple rewiring rules can indeed support memory protection, we performed simulations as described above. Input assembly activations and background noise were generated as before, however, assemblies were activated in a sequential manner. In other words, we first activated exclusively assembly A_1_ 250 times, then assembly A_2_, then assembly A_3_, etc. (with a delay between each pattern presentation as in the previous simulations; Figure 4A). Figure 4B shows schematic drawings of the 12 simulated branches at various times of the rewiring process. The color of a branch in Figure 4B indicates which of the 8 sequentially presented assemblies had evolved a synaptic cluster at the branch at the specified time. Clearly, branches were recruited sequentially to store input patterns. Branch b_2_ received a synaptic cluster from the first assembly, whereas none of the other branches was evolving large weights for this assembly. Beginning at 125 s, assembly A_2_ was activated while assembly A_1_ was silent. Shortly afterwards, branch b_4_ developed large synaptic weights for this input pattern while the synapse cluster at branch b_2_ remained stable. This stabilization is due to the fact that (a) synapses from assembly A_2_ to branch b_2_ were weak, and dendritic plateau potentials were consequently induced rarely, and (b), presynaptic activity from assembly A_1_ was weak, and depression due to inverse STDP was thus very small. As further assemblies became activated every 125 s, new branches were recruited to store these patterns while synaptic weights on old branches kept stable. In this simulation, all assemblies were represented on the branches. Four branches remained without a synapse cluster from any of the assemblies. We have repeated the experiment for 25 independent trials. The mean number of represented assemblies over these trials was 6.92 ± 0.89 (mean ± SD).

We hypothesized that competition between branches through STDP is essential for this capability. Due to the competition, only one or a few branches evolve a synaptic cluster from a specific assembly while all other branches remain neutral to assemblies. This is especially important when assemblies are presented in a sequential manner. Without competition, most of the branches would adapt to respond to the first active assembly. Most branches would thus be occupied and not available for novel assemblies. To quantify the effect of dendritic competition, we repeated the experiment without STDP. In this case, the mean number of represented assemblies reduced to 4.04 ± 0.72 (mean ± SD over 25 independent trials). As expected, most of the branches evolved synaptic clusters from the first shown assembly A_1_ early on.

Due to the random fluctuations of weights, there is always a chance that synapses are overwritten. This behavior can be interpreted as gradual forgetting. If a synaptic cluster is inactive for an extended time, then the synaptic connections gradually degrade. If, however, that assembly is again activated, the synapses to that assembly will again stabilize provided that the weights are large enough to trigger a dendritic spike. To investigate the impact of the random fluctuations of weights in the weakening of older memories over time, we have performed simulations with various noise strengths and analyzed the weight decrease (see section 1.4 in the Supplementary Material for more detail). In section 1.5 of the Supplementary Material, we show a simple way to reduce gradual forgetting in our model and in section 1.6 of the Supplementary Material we propose a simple consolidation mechanism. Both of these methods integrate nicely with our rewiring framework. Synaptic connections of old memories are well-protected against changes induced by the noise term in this extended model (see sections 1.5, 1.6 in the Supplementary Material).

### 2.5. Synaptic Clustering Depends on Input Statistics

In the above simulations, input assemblies were activated in temporal isolation and each assembly activation was clean in the sense that all assembly neurons had an elevated firing rate and assemblies were disjoint. Under these conditions, our rewiring model led to robust synaptic clustering. We next determined the sensitivity of this result on these input statistics.

**Figure 5:**
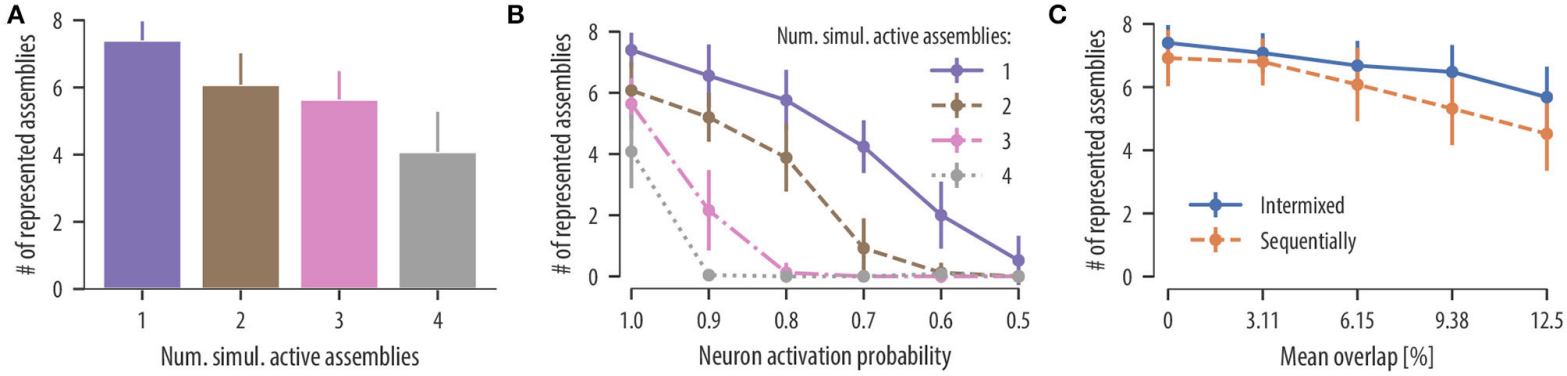
Synaptic clustering depends on input statistics. **(A)** Mean number of represented assemblies as a function of the number of simultaneous active assemblies. The difference in the mean number of represented assemblies for two and three simultaneous active assemblies were not significant. However, the number of represented assemblies significantly decreased compared to temporally isolated pattern presentations in all cases [*F*_(3, 96)_ = 53.9, *p* < 0.0001, one-way analysis of variance (ANOVA); *p* < 0.0001, one active assembly vs. two simulations active assemblies, 1 vs. 3, 1 vs. 4, 2 vs. 4, 3 vs. 4; not significant (ns) *p* = 0.35, 2 vs. 3; *n* = 25 trials]. These results show that the rewiring mechanism segregates uncorrelated input assemblies onto different branches even when a medium number of assemblies are activated simultaneously. **(B)** Mean number of represented assemblies as a function of the assembly neuron activation probability (mean and SD over 25 independent trials). Results are shown for temporally isolated pattern presentations (violet solid line), two simultaneous active assemblies (brown dashed line), three simultaneous active assemblies (pink dashed dotted line), and four simultaneous active assemblies (gray dotted line). In all four cases, the number of represented assemblies significantly decreased at a neuron activation probability of 0.9 [*t*_(24)_ = 5.09, *p* < 0.0001, one active assembly, activation probability of 1.0 vs. activation probability of 0.9; *t*_(24)_ = 4.99, *p* < 0.0001, two active assemblies; *t*_(24)_ = 10.8, *p* < 0.0001, three active assemblies; *t*_(24)_ = 19.2, *p* < 0.0001, four active assemblies, paired *t*-test]. **(C)** Mean number of represented assemblies as a function of the mean overlap between a given pair of assemblies (mean and SD over 25 independent trials). Results are shown for intermixed pattern presentations (blue solid line) and for patterns presented in a sequential manner as in Figure 4 (orange dashed line). In both cases, the number of represented assemblies significantly decreased for a mean assembly overlap of 6.15 % [*t*_(24)_ = 3.84, *p* = 0.0008, intermixed, disjoint assemblies vs. non-disjoint assemblies with mean overlap of 6.15 %; *t*_(24)_ = 2.87, *p* = 0.008, sequentially, paired *t*-test].

*Influence of co-active assemblies:* We first asked whether input assemblies can be clustered onto different dendritic compartments even when single assemblies are never activated in temporal isolation. As above, we simulated a neuron with 12 independent branches and a population of 320 input neurons divided into 8 disjoint neuronal assemblies of 40 neurons each. Input patterns and background noise were generated as before. At times of assembly activation, a medium number of assemblies was chosen uniformly at random from the set of all input assemblies and were simultaneously activated for 300 ms. For up to four simultaneous active assemblies, the rewiring dynamics separated connections from different assemblies onto different branches with some decrease of clustering. In 25 independent simulation runs, the mean number of represented assemblies on the neuron was 6.08 ± 0.94 for two simultaneously activated assemblies, 5.64 ± 0.84 for three simultaneously activated assemblies, and 4.08 ± 1.20 for four simultaneously activated assemblies (Figure 5A). Our rewiring mechanisms consequently segregates uncorrelated inputs onto different branches even when assembly patterns are not temporally isolated.

*Influence of stochastic assembly activation:* While evidence for synaptic clustering has been reported in various cortical areas (Chen et al., 2011; Kleindienst et al., 2011; Fu et al., 2012), some studies found no evidence for it in early sensory areas (Jia et al., 2010). We hypothesized that this discrepancy may be contributed to different input statistics in different cortical areas. In particular, we conjectured that inputs to neurons in early sensory areas may be more stochastic (or unreliable) than in higher cortical areas. To study this issue quantitatively, we investigated how clustering in our model depends on the reliability of assembly activations. To this end, we repeated the experiment with simultaneously activated assemblies but with a reduced number of active neurons in each assembly activation. When an assembly was activated for 300 ms, a fraction *p* of assembly neurons was chosen randomly which increased their firing rate to 35 Hz as before, while the rest remained at the background rate of 1 Hz. In successive simulations, we reduced the fraction *p* of active neurons in each assembly from 1.0 (all neurons were active at each pattern presentation) down to 0.5 (half of the neurons were active at each pattern presentation). The mean number of represented assemblies as a function of the neuron activation probability in each assembly is shown in Figure 5B (25 independent trials). The number of assembly-synapse clusters on dendritic branches decreased with the neuron activation probability. A moderate stochasticity is tolerated by the rewiring dynamics for temporally isolated patterns and two simultaneously activated assemblies. No stochasticity is tolerated, however, when more than two assemblies are activated simultaneously at times of pattern presentation (Figure 5B). In all simulations, a number of branches did not receive any synaptic cluster. This was expected as the neuron had 12 branches while only 8 assembly patterns were presented as input. These branches hosted rather stable synaptic connections originating from various input assemblies. In conclusion, this result predicts a continuum of synaptic clustering in PCs where the extent of synaptic clustering depends on the reliability of presynaptic assembly activations. This finding may provide an explanation for diverse results on synaptic clustering in cortex.

*Influence of assembly overlap:* In the simulations so far, we considered disjoint input assemblies. In contrast, it was proposed that associative memories learned within short time intervals will be represented by overlapping populations of neurons (Silva et al., 2009; Rogerson et al., 2014). This claim was recently verified by experimental studies of medial temporal lobe (Ison et al., 2015), the hippocampus (Cai et al., 2016), and the lateral amygdala (Rashid et al., 2016). Their results show that linking of memories relies on the overlap of distributed neuronal assemblies.

To test whether synaptic clustering emerges also for input assemblies with various amounts of neuron overlap, we considered a setup where some of the neurons of each assembly were chosen from a shared pool of neurons. As a result, some neurons of the shared pool participated in two or more input assemblies (see section 4 for details). By increasing the fraction of shared neurons in the assemblies, we were able to increase the assembly overlap. When repeating this procedure in 25 independent trials, the overlap between a given pair of assemblies was 3.11 ± 2.48% (mean ± SD), 6.15 ± 3.53%, 9.38 ± 4.19%, and 12.5 ± 5.11% for a fraction of shared neurons per input assembly of 25, 50, 75, and 100%, respectively. After 17 min of assembly activations, we determined the number of represented assemblies on the neuron. The results for 25 independent trials are summarized in Figure 5C (solid line). Our results show that synapses are organized on dendrites in a clustered manner; this is true even for assemblies that share a significant portion of neurons, where the number of represented assemblies decreases slightly with increasing assembly overlap.

In section 2.4, we showed that our rewiring rule can support memory protection by recruiting branches sequentially as new assemblies were activated. To test whether this ability of the model depends on disjoint input assemblies, we repeated the experiment described in section 2.4 but with overlapping assemblies and various assembly overlaps. Overlapping assemblies were created as described above. After 17 min of assembly activations, the mean number of represented assemblies was 6.80 ± 0.75, 6.08 ± 1.16, 5.32 ± 1.16, and 4.52 ± 1.17 for mean assembly overlaps of 3.11 ± 2.48%, 6.15 ± 3.53%, 9.38 ± 4.19%, and 12.5 ± 5.11%, respectively (mean and SD over 25 independent trials). For all considered overlaps, synapses were still segregated in an assembly-specific manner. However, the number of represented assemblies significantly decreased for mean assembly overlaps of 6.15 % (Figure 5C, dashed line). In summary, our model predicts that associated memories with overlapping assembly representations are segregated in an assembly-specific manner onto different dendritic branches and that this segregation can indeed support memory protection.

### 2.6. Analysis of Stochastic Rewiring Dynamics

The stochastic rewiring dynamics considered in this work is described by the general SDE (1), reiterated here for convenience:

(3) $$\begin{array}{l} d\theta_{ki}\left(t\right)=\eta \left(f_{ki}^{S}\left(t\right)+f_{ki}^{L}\left(t\right)\right)dt+\sqrt{2\eta T}dW_{ki}, \end{array}$$

where the first term $f_{ki}^{S}$ describes structural constraints on the connectivity, the second term $f_{ki}^{L}$ captures the functional goal of the plasticity process, and the third term models stochastic contributions.

It has been shown by Kappel et al. (2015) that the long-term behavior of this dynamics can be described concisely if the deterministic parameter changes are defined through two probability distributions:

(4) $$\begin{array}{l} f_{ki}^{S}\left(t\right)=\frac{\partial }{\partial \theta_{ki}}\log p_{S}\left(\theta \right){|}_{\theta \left(t\right)}\;\text{and }f_{ki}^{L}\left(t\right)=\frac{\partial }{\partial \theta_{ki}}\log p_{L}\left(\theta \right){|}_{\theta \left(t\right)}, \end{array}$$

where $p_{S}$ is a probability distribution that defines the structural constraints (the *structural prior* on parameters), and $p_{L}$ is a distribution that defines functionally preferred parameter settings (the *functional likelihood* of parameters). Preferred parameter settings have high probability in these distributions, others have low probability. Note that without the noise term, Equation (1) together with Equation (4) constitute continuous-time gradient ascent dynamics on the objective $\log p_{S}\left(\theta \right)+\log p_{L}\left(\theta \right)=\log\left(p_{S}\left(\theta \right)p_{L}\left(\theta \right)\right)$. With the inclusion of the noise term, Equation (1) defines Langevin sampling dynamics (Welling and Teh, 2011), that is, parameter configurations are visited in the long run according to the distribution

(5) $$p^{*}(\theta )\propto {\left(p_{S}(\theta )p_{L}(\theta )\right)}^{1/T}.$$

In other words, the probability density to observe some specific **θ** is given—after an initial transient period (often called burn-in time) where parameters move from the potentially low-probability initial setting to a high-probability region—by p^*^(**θ**). We say that the network samples network configurations from p^*^(**θ**). Since this p^*^(**θ**) contains the product of the two distributions $p_{S}$ and $p_{L}$, high-probability parameter settings are only possible if they fulfill both the structural and the functional demands as defined by these distributions. Since **θ** defines both the synaptic strengths and the connectivity structure (a synapse *ki* is connected if θ_*ki*_ > 0), this stochastic process automatically samples network connectivity structures.

The structural prior corresponding to the structural plasticity term $f_{ki}^{S}$ is discussed in detail in section 4. The functional term $f_{ki}^{L}\left(t\right)$ is given by

(6) $$f_{ki}^{L}(t)=\{\begin{array}{ll} c_{L}\Gamma_{k}(t)(x_{i}(t)-\gamma (1-x_{i}(t))), & \text{if }\theta_{ki}(t)>0\\ \; & \text{(functional connection)}\\ 0, & \text{if }\theta_{ki}(t)\leq 0\\ \; & \text{(non-established}\\ \; & \text{connection)}. \end{array}$$

Synapses that contribute or are co-active with dendritic plateau potentials are potentiated, while others are depressed and eventually retract, making room for new connections.

To interpret this functional term from the perspective of the sampling distribution of the stochastic dynamics, we want to exhibit a corresponding functional likelihood $p_{L}$. We show in section 4 that this distribution factors into branch-specific distributions

(7) $$\begin{array}{l} p_{L}\left(\theta \right)=\underset{k}{\prod }p_{L}\left(\theta_{k}\right), \end{array}$$

so each branch optimizes its likelihood independently from other branches. For the functional likelihood, we consider the situation where the neuron is exposed for an extended time *t*_max_ to input spike trains *x*_*i*_ according to some distribution and it stochastically responds with branch spikes. We can interpret the functional dynamics using the following functional likelihood (see section 4)

(8) $$\begin{array}{l} p_{L}(\theta_{k})=\\ \;\frac{1}{Z_{L}}\exp{\langle \frac{{\hat{c}}_{L}}{t_{\text{max}}}\int_{0}^{t_{\text{max}}}\Gamma_{k}(s)\left(\sum_{i}w_{ki}x_{i}(s)-\gamma \sum_{i}w_{ki}(1-x_{i}(s))\right)ds\rangle }_{\;X,\Gamma_{k}}, \end{array}$$

where $Z_{L}$ is a normalizing factor (we consider here parameters bounded in the range [θ_min_, θ_max_] to keep this factor bounded) and ${ĉ}_{L}>0$ is a constant. The angular brackets 〈·〉_*X*, Γ_ denote an average over stochastic realizations of inputs *x*_*i*_ and branch spikes Γ_*k*_. Because of the function Γ_*k*_ in the integral, only times at which a plateau potential is present at the branch contribute to the integral. In combination with the first term in the brackets, the probability of the functional likelihood is high if efficacies of those synapses are large that are active slightly before or during a plateau potential. In combination with the second term in the brackets, the probability of the functional likelihood is high if efficacies of those synapses are small that are inactive slightly before or during a plateau potential. The constant γ > 0 trades off the importance of large active vs. small inactive synapses.

Hence, the rewiring dynamics prefers parameter settings where inputs that jointly recruit dendritic regenerative events (i.e., temporally correlated inputs) have strong synaptic connections to the branch. Under the assumption that such inputs are also functionally related (corresponding for example to a specific motor behavior), the rewiring dynamics contributes to functional synaptic clustering (Kastellakis et al., 2015). While cortical connectivity is sparse even at the local scale, it has been estimated that potential (i.e., possibly realizable) connectivity is huge—a spatial scale of a few hundred microns—and every pair of PCs could be potentially connected (Chklovskii et al., 2004; Kalisman et al., 2005). Our analysis shows that in this large space of potential connectivity structures, synaptic rewiring based on simple dendritic-spike-dependent plasticity rules could implement a stochastic search algorithm that favors functionally clustered synaptic configurations, as proposed by Chklovskii et al. (2004).

## 3. Discussion

We have proposed in this article a model for synaptic rewiring in neurons with dendritic non-linearities. We have observed that simple plasticity rules that depend on postsynaptic dendritic spikes lead to robust synaptic clustering. The use of the synaptic sampling framework allowed us to analyze the connectivity dynamics as a sampling process from a distribution of network configurations. Our analysis revealed that synaptic clusters are preferred configurations in this sampling process. Cichon and Gan (2015) found that dendritic spike events for different motor tasks were segregated onto different tuft branches of layer V PCs in mouse motor cortex. They also found that this segregation was necessary to avoid a detrimental effect of training on a previously learned task. Our model can explain these findings. First, we found that branch activity is segregated onto different dendritic branches if it is elicited by different input assemblies. Second, we found that this segregation allows memory retention in the sense that synaptic clusters are retained after memorization of new assembly input patterns.

### 3.1. Synaptic Plasticity and Rewiring

Throughout this article, we assumed that strong presynaptic activity coincident with a postsynaptic dendritic spike leads to LTP whereas a postsynaptic dendritic spike paired with weak presynaptic activity leads to LTD. While these plasticity dynamics are qualitatively consistent with experimental findings both for LTP (Golding et al., 2002; Lisman and Spruston, 2005; Gordon et al., 2006; Gambino et al., 2014; Cichon and Gan, 2015; Brandalise et al., 2016) and LTD (Holthoff et al., 2004), the exact plasticity rules and their relation to dendritic regenerative events are still to be determined. For example, experimental results of a study by Cichon and Gan (2015) indicated that at distal dendrites of layer V PCs, presynaptic activity during dendritic spikes leads to LTP, which is consistent with our model. However, different from previous findings, they found that LTD was induced by presynaptic activity that preceded the dendritic spike. To test whether our model is sensitive to such alterations in the plasticity rules, we reran the simulations for Figure 3 with such depression (see section 4 for details). We found that the model behaved comparably (7.28 ± 0.60 represented assemblies, mean ± SD over 25 independent trials).

We found that the number of input patterns that can be stored by a neuron is increased if dendritic-spike-dependent plasticity is extended by an STDP mechanism where pre-before-post spike pairs induce depression. This phenomenon has already been described and analyzed in a modeling study by Legenstein and Maass (2011). In general, the back-propagating action potential provides a means for communicating global information to the dendritic branches, that is, it can indicate to all branches that the neuron is active for the current input pattern. With STDP, a pattern that does already activate enough branches to elicit strong firing of the neuron will depress synapses at inactive branches. This competition reduces the number of activated branches per pattern and thus increases the number of patterns that can be stored in a single neuron. While we showed that clustering is still observed without this mechanism (Figure 2), it is beneficial in a sequential learning paradigm, as it keeps branches free to store patterns presented later in the sequence (Figure 4).

### 3.2. Protection of Memories

Our results indicate that synaptic clustering may protect older memories (Figure 4). It can thus avoid catastrophic forgetting, a well-known deficiency of standard incremental learning algorithms for neural networks (McCloskey and Cohen, 1989; French, 1999; Kirkpatrick et al., 2017). The mechanism is simple: a branch with a synaptic cluster from a specific input pattern is silent to most other patterns. Since LTP and LTD is conditioned on the local dendritic spike, memories on a branch are protected for most other input patterns. At the same time, other branches can develop specificity to novel patterns as plasticity there is decoupled from protected branches. However, this does not shelter memories completely. There are still mechanisms in our model that are detrimental to old memories. In particular, the noise term in Equation (1) will inevitably corrode memories. We did indeed observe weakening of older memories over time if they were not repeated, but on a time scale much longer than the one of pattern presentation (sections 1.4, 1.5 in the Supplementary Material). This suggests that synaptic clustering may provide a mechanism to avoid catastrophic forgetting temporarily. Additional consolidation mechanisms seem necessary to protect memories over long time scales, and we have investigated one way how consolidation can be integrated in our rewiring framework (section 1.6 in the Supplementary Material). An alternative role of dendritic structures in extending memory lifetime was proposed in Bono and Clopath (2017). We have demonstrated in this article that simple dendritic-spike-dependent rewiring can protect memories in a pattern memorization setup. In future work, one could apply these ideas to larger deep-learning networks in the context of continual learning tasks, possibly using simplified neuron and plasticity models with similar properties.

### 3.3. Related Work

Many theoretical studies have linked rewiring processes to cortical connection statistics (Deger et al., 2012; Fauth et al., 2015), computation and memory (Knoblauch et al., 2014; Zheng and Triesch, 2014; Deger et al., 2016; Miner and Triesch, 2016; Spiess et al., 2016), and homeostasis (Gallinaro and Rotter, 2018). These studies were, however, based on point neuron models, where the phenomenons considered in the current study cannot be observed. It has been proposed by Rhodes (2008) that neurons with non-linear dendrites could serve as a recoding stage in order to orthogonalize input representations, that is, to reduce the overlap between different input patterns. It is discussed there that a dendritic-spike-dependent rewiring could be used to provide efficient wiring diagrams for this operation. Our model exhibits one possible implementation of such a rewiring strategy. In a theoretical study, the impact of active dendrites on the memory capacity of neural networks was investigated (Poirazi and Mel, 2001). They calculated that non-linear dendrites can vastly increase the number of patterns that can be stored by a simple non-spiking model of a pyramidal cell. They also considered a supervised structural plasticity rule, the “clusteron” (Mel, 1992), that was designed to cluster synaptic inputs while optimizing the memory performance of the neuron. Their simulations showed that this rule leads to memory capacities as predicted by their theoretical considerations. Our model shows that such clustering emerges also in a more detailed spiking neuron model with a simpler online dendritic-spike-dependent rewiring principle that does not need supervision. We exhibited a potential functional relevance of this clustering in terms of protection of memory but did not consider memory capacity *per se*. It would be interesting to study similar capacity questions in the proposed model. Memory capacity of networks of neurons with non-linear dendrites was also studied by Wu and Mel (2009). They also used a simple non-spiking neuron model which basically consisted of a “dendritic” layer with a threshold non-linearity and a somatic layer which summed the outputs of the binary dendritic elements. This article did not consider rewiring. Nevertheless, a simple plasticity rule was used that depended on the dendritic activation, which resembles some similarity with dendritic-spike-dependent plasticity considered here. Interestingly, they found that memory capacity could be increased if not only the dendritic activation but also the number of activated synapses at the dendrite was used to gate LTP. In our rewiring framework, such a rule could be applied to weak synaptic connections, but not to non-established connections as their dynamics are assumed independent of pre- and postsynaptic activity.

### 3.4. Relation to Experimental Literature and Experimentally Testable Predictions

The proposed model gives rise to a number of experimentally testable predictions. We have shown that temporally correlated input leads to the clustering of corresponding synaptic connections on single dendritic branches. This suggests that neuronal assemblies should connect to PCs preferentially in a branch-specific clustered manner. To verify this prediction, methods for labeling memory engrams (Tonegawa et al., 2015) could either be combined with tracing techniques or with engram reactivation and target cell Ca^2+^ imaging at dendritic branches. Another prediction of our model is that synaptic clustering depends on input statistics. This finding may explain that no evidence for clustering has been observed in early sensory cortical areas (Jia et al., 2010). One could test this prediction more directly through glutamate uncaging at tuft dendrites. Our model predicts that concerted correlated uncaging at single branches should lead to a clustered strengthening of synapses at the branch. Conversely, a more unreliable uncaging where each uncaging site is activated only with a certain probability would not have this effect. Various experimental studies have indicated that associated memories are stored in overlapping assemblies (Ison et al., 2015; Cai et al., 2016; Rashid et al., 2016). Our model predicts that such associations should also be visible in the clustering structure, that is, neurons that respond to each of two associated memories would exhibit synaptic co-clustering of these memories within dendrites. This prediction is consistent with the findings of a modeling study by Kastellakis et al. (2016).

### 3.5. Conclusions

We have shown through computer simulations and theoretical analysis that synaptic clustering emerges from simple rewiring dynamics based on dendritic-spike-dependent synaptic plasticity. This result provides support for the synaptic clustering hypothesis. We further found that this clustering in turn may serve as a mechanism to protect memories from subsequent modifications on a medium time scale. More generally, our results emphasize the importance of dendritic structures in learning and memory and demonstrate the effectiveness of synaptic rewiring to find sparse network structures in the high-dimensional search space over potential connectivities.

## 4. Materials and Methods

Here, we provide details to the models and simulations.

### 4.1. Neuron Model

We assume a spiking neuron model with *N*_b_ independent dendritic compartments. Here and in the following, we will indicate variables and parameters of dendritic branches with a b superscript or subscript. Presynaptic spikes are transformed at the synapse *ki*—the synapse from presynaptic neuron *i* to branch *k* of the postsynaptic neuron—into alpha-shaped postsynaptic currents, which are weighted by the corresponding synaptic efficacies *w*_*ki*_. The total current at branch *k* is given by

(9) $$\begin{array}{l} I_{k}^{\text{b}}\left(t\right)=\underset{i}{\sum }w_{ki}\underset{f}{\sum }\alpha \left(t-t_{\text{pre},i}^{\left(f\right)}\right) \end{array}$$

where $t_{\text{pre},i}^{\left(f\right)}$ is the *f*^th^ spike-time of presynaptic neuron *i*. The two sums run over all presynaptic neurons *i* and all firing times $t_{\text{pre},i}^{\left(f\right)}<t$ of neuron *i*. Each individual alpha-current is given by

(10) $$\begin{array}{l} \alpha \left(s\right)=\frac{s}{\tau_{\text{syn}}}\exp\left(1-\frac{s}{\tau_{\text{syn}}}\right)H\left(s\right), \end{array}$$

where *H* denotes (here and in the following) the Heaviside step function (for parameter values, see Table 1). The membrane potential of each branch ($V_{k}^{\text{b}}$; branch potential) follows the dynamics of a leaky integrator of the current with resting potential $E_{\text{L}}^{\text{b}}$. In particular, the dynamics of potential $V_{k}^{\text{b}}$ of branch *k* is given by

(11) $$\begin{array}{l} C_{\text{b}}\frac{dV_{k}^{\text{b}}\left(t\right)}{dt}=-\frac{1}{R_{\text{b}}}\left(V_{k}^{\text{b}}\left(t\right)-E_{\text{L}}^{\text{b}}\right)+I_{k}^{\text{b}}\left(t\right), \end{array}$$

with membrane resistance *R*_b_ and membrane capacity *C*_b_ (the membrane time constant is given by τ_b_ = *R*_b_*C*_b_).

**Table 1:** Neuron parameters for computer simulations.

**Branch parameters**		
$V_{\text{th}}^{\text{b}}$	Stochastic threshold	-55 mV
$E_{\text{L}}^{\text{b}}$	Leak reversal potential	-70 mV
*R*_b_	Membrane resistance	40 MΩ
*C*_b_	Membrane capacity	250 pF
τ_s_	Time constant of sodium spikelet	4 ms
*V*_ds_	Amplitude of plateau potential	-30 mV
*V*_s_	Initial amplitude of sodium spikelet (rel. to *V*_ds_)	5 mV
*c*_ds_	Scale factor for plateau duration	0.04 s^2^/mV
$\rho_{0}^{\text{b}}$	Instantaneous firing rate at threshold	2.5 Hz
β^b^	Sensitivity of spike emission	0.5 mV^-1^
$\Delta_{\text{min}}^{\text{ds}}$	Minimum duration of a plateau potential	20 ms
$\Delta_{\text{max}}^{\text{ds}}$	Maximum duration of a plateau potential	300 ms
**Soma parameters**		
$V_{\text{th}}^{\text{soma}}$	Stochastic threshold	-55 mV
$E_{\text{L}}^{\text{soma}}$	Leak reversal potential	-70 mV
*R*_m_	Membrane resistance	40 MΩ
*C*_m_	Membrane capacity	250 pF
*R*_l_	Longitudinal resistance along dendrites and apical trunk	2Ω
$\Delta_{\text{abs}}^{\text{soma}}$	Absolute refractory period	5 ms
$\rho_{0}^{\text{soma}}$	Instantaneous firing rate at threshold	2.5 Hz
β^soma^	Sensitivity of spike emission	0.5 mV^-1^
**Synapse parameters**		
$\tau_{\text{syn}}^{\text{b}}$	Synaptic time constant of branch	2 ms
θ_min_	Lower bound of synaptic parameter	-2
θ_max_	Upper bound of synaptic parameter	8

Branch spikes are elicited by the branch in a stochastic manner. In particular, the voltage-dependent instantaneous firing rate $f_{k}^{\text{b}}\left(t\right)$ is given by

(12) $$f_{k}^{\text{b}}(t)=\{\begin{array}{ll} \rho_{0}^{\text{b}}\exp\left(\beta^{\text{b}}(V_{k}^{\text{b}}(t)-V_{\text{th}}^{\text{b}})\right), & \text{if }\frac{dV_{k}^{\text{b}}(t)}{dt}>0\\ 0, & \text{else}, \end{array}$$

where $V_{\text{th}}^{\text{b}}$ is the threshold for branch-spike initiation, $\rho_{0}^{\text{b}}$ is the instantaneous firing rate at threshold, and β^b^ is the sensitivity of spike emission (for parameter values, see Table 1). We denote the times of branch-spike onsets at branch *k* as $t_{k}^{\left(1\right)},t_{k}^{\left(2\right)},\ldots \;$. A branch spike includes a brief sodium spikelet followed by a plateau potential that lasts for up to a few hundreds of milliseconds, see Figure 1B. Assuming that branch *k* has initiated its last dendritic spike at time ${\hat{t}}_{k}\left(t\right)$, the evolution of $V_{k}^{\text{b}}\left(t\right)$ immediately after initiation is given by

(13) $$\begin{array}{l} V_{k}^{\text{b}}\left(t\right)=\eta_{k}\left(t-{\hat{t}}_{k}\left(t\right)\right)\;\text{for }{\hat{t}}_{k}\left(t\right)<t\leq {\hat{t}}_{k}\left(t\right)+\Delta_{k}^{\text{ds}}\left({\hat{t}}_{k}\left(t\right)\right), \end{array}$$

where the function η_*k*_ describes the form of the branch spike (see below). The duration of the dendritic spike is proportional to the slope of $V_{k}^{\text{b}}\left(t\right)$ at the last spike time of the branch, i.e.,

(14) $$\begin{array}{l} \Delta_{k}^{\text{ds}}\left({\hat{t}}_{k}\left(t\right)\right)=c_{\text{ds}}\frac{dV_{k}^{\text{b}}\left(t\right)}{dt}{|}_{t={\hat{t}}_{k}\left(t\right)}, \end{array}$$

where *c*_ds_ is a scaling parameter. In our simulations, we clipped the duration between a minimum of $\Delta_{\text{min}}^{\text{ds}}=\text{20}\;\text{ms}$ and a maximum of $\Delta_{\text{max}}^{\text{ds}}=\text{300}\;\text{ms}$ to avoid unnaturally brief and long plateau durations (for other parameter values, see Table 1). The functional relation between input strength and the duration of the plateau potential $\Delta_{k}^{\text{ds}}\left({\hat{t}}_{k}\left(t\right)\right)$ is shown in Figure 1C. The shape of the dendritic spike is given by a brief sodium spikelet followed by a plateau (Antic et al., 2010):

(15) $$\begin{array}{l} \eta_{k}\left(s\right)=V_{\text{s}}\exp\left(-\frac{s}{\tau_{\text{s}}}\right)H\left(s\right)+V_{\text{ds}}\left[H\left(s\right)-H\left(s-\Delta_{k}^{\text{ds}}\left({\hat{t}}_{k}\left(s\right)\right)\right)\right]. \end{array}$$

The first term in Equation (15) models the sodium spikelet, and the second term models the plateau potential. The sodium spikelet decays exponentially with time constant τ_s_ with initial amplitude *V*_s_ (added to the amplitude *V*_ds_ of the plateau potential). For $t>{\hat{t}}_{k}\left(t\right)+\Delta_{k}^{\text{ds}}\left({\hat{t}}_{k}\left(t\right)\right)$ the membrane potential $V_{k}^{\text{b}}\left(t\right)$ again follows the dynamics given by Equation (11). As an example, the evolution of the dendritic membrane potential for an arbitrary chosen input is depicted in Figure 1B.

During the time of the plateau potential, the dendritic branch is absolute refractory, that is, no further regenerative event can be initiated. The propagation of dendritic plateau potentials toward the soma produces a sustained depolarization of the cell body. In general, the currents from dendritic compartments to the soma are driven by the difference between the membrane potential at the branch and the membrane potential of the soma. The somatic compartment sums currents from all dendrites (with a certain degree of amplitude attenuation).

The membrane potential at the soma of the neuron is given by

(16) $$\begin{array}{l} C_{\text{m}}\frac{dV^{\text{soma}}\left(t\right)}{dt}=-\frac{1}{R_{\text{m}}}\left(V^{\text{soma}}\left(t\right)-E_{\text{L}}^{\text{soma}}\right)+I^{\text{soma}}\left(t\right), \end{array}$$

where $E_{\text{L}}^{\text{soma}}$ is the resting potential. The membrane time constant of the neuron is given by τ_m_ = *R*_m_*C*_m_ and *I*^soma^(*t*) is

(17) $$\begin{array}{l} I^{\text{soma}}\left(t\right)=\frac{1}{R_{\text{l}}}\underset{k}{\sum }\max\left\{0,V_{k}^{\text{b}}\left(t\right)-V^{\text{soma}}\left(t\right)\right\}, \end{array}$$

where *R*_l_ represents the total of the longitudinal resistance along the dendrites and apical trunk. Note that we restrict the currents to non-negative values since we only simulate forward currents, i.e., currents from the dendritic branches to the soma. Somatic spikes are characterized by their spike times *t*^(*f*)^. The instantaneous firing rate is given analogously to the branch spike mechanism by

(18) $$f^{\text{soma}}(t)=\{\begin{array}{ll} \rho_{0}^{\text{soma}}\exp\left(\beta^{\text{soma}}(V^{\text{soma}}(t)-V_{\text{th}}^{\text{soma}})\right), & \text{if }\frac{dV^{\text{soma}}(t)}{dt}>0\\ 0, & \text{else}. \end{array}$$

Immediately after *t*^(*f*)^, the somatic potential enters a refractory period of duration $\Delta_{\text{abs}}^{\text{soma}}$ in which *V*^soma^(*t*) is set to $E_{\text{L}}^{\text{soma}}$. For $t>t^{\left(f\right)}+\Delta_{\text{abs}}^{\text{soma}}$ the dynamics is again given by Equation (16). To illustrate the dynamics of the membrane potential, a neuron which receives arbitrary input was simulated. The results of this simulation are shown in Figure 1B.

### 4.2. Plasticity and Rewiring

Here, we summarize the plasticity and rewiring dynamics in the network.

#### 4.2.1. Synaptic Parameters and Efficacies

Each dendritic compartment has a set of potential synaptic connections that could be realized by some parameter setting. However, at each point in time, only a subset of these connections is realized by functional synapses. More precisely, we maintain one parameter θ_*ki*_ for each synapse *ki* from input neuron *i* to branch *k* of the neuron. This parameter encodes (a) whether the synapse is functional and (b) the synaptic efficacy *w*_*ki*_ of the synapse (if it is functional). In particular, θ_*ki*_ > 0 indicates a functional synapse, while θ_*ki*_ ≤ 0 indicates that the connection is not established. The synaptic weight *w*_*ki*_ is *c*_θ_θ_*ki*_ for a functional synapse, and 0 otherwise. Hence, the mapping from the parameter θ_*ki*_ to the synaptic weight is given by *w*_*ki*_ = *c*_θ_max{0, θ_*ki*_} where *c*_θ_ = 1 nA is the slope of this mapping for θ_*ki*_ > 0 (for other parameter values, see Table 2).

**Table 2:** Plasticity parameters for computer simulations.

η	Learning rate	0.002
*T*	Temperature parameter	0.3
$c_{L}$	Scale factor of functional plasticity term	1.5
γ	Scale factor for depression of functional plasticity term	0.2
λ	Scale factor for steepness of sigmoid in structural plasticity term	10
*c*_w_	Scale factor for steepness of sigmoid in synapse count	0.55 nA^-1
*c*_θ_	Slope of θ to *w* mapping (for θ > 0)	1 nA
$c_{\mathrm{STDP}}$	STDP, LTD factor	3.2
${\mathrm{STDP}}_{\text{th}}$	Threshold for STDP initiation	-67 mV
τ_x_	Time constant of presynaptic trace	20 ms

#### 4.2.2. Parameter Dynamics

The stochastic dynamics of parameters are given by

(19) $$\begin{array}{l} d\theta_{ki}\left(t\right)=\eta \left(f_{ki}^{S}\left(t\right)+f_{ki}^{L}\left(t\right)\right)dt+\sqrt{2\eta T}dW_{ki}, \end{array}$$

where η > 0 is a small learning rate and $W_{ki}$ denote increments of a standard Wiener process, thus implementing random walk behavior. The temperature *T* > 0 is a constant that scales the strength of this stochastic component. In the simulations, the parameters θ_*ki*_ were clipped to the range [θ_min_, θ_max_]. The upper bound is necessary to avoid that the synaptic efficacies become arbitrary large. The lower bound is useful to avoid that parameters become very negative. Such parameters would be stuck for a long time in the negative domain and hence would be unlikely to be re-established, which would slow down the rewiring dynamics. The term in the brackets defines the deterministic part of the dynamics. It is divided here into two components. The first term $f_{ki}^{S}$ describes changes that are used to enforce structural constraints on the connectivity. The functional goal of the plasticity process is captured by the second term $f_{ki}^{L}$. The deterministic terms are chosen such that they vanish for θ_*ki*_(*t*) ≤ 0. Hence, for non-established synapses, only the stochastic term remains.

For the structural term, we define a soft count of synaptic connections on branch *k*

(20) $$\begin{array}{l} N\left(\theta_{k}\right)=\underset{i}{\sum }2\left(\sigma \left(c_{\text{w}}w_{ki}\right)-\frac{1}{2}\right), \end{array}$$

where σ denotes the logistic sigmoid function $\sigma \left(x\right)=\frac{1}{1+\exp\left(-x\right)}$. For a non-established synapse (*w*_*ki*_ = 0), the term in the brackets is 0 and this potential connection is not counted. For increasing efficacies larger than 0, this term approaches 1 with the scaling constant *c*_w_ defining the speed of this approach. Using this soft count, the structural term is given by

(21) $$\begin{array}{l} f_{ki}^{S}\left(t\right)=-2\lambda c_{\text{w}}c_{\theta }\left[1-\sigma \left(\lambda \left(N_{\text{syn}}-N\left(\theta_{k}\left(t\right)\right)\right)\right)\right]\dot{\sigma }\left(c_{\text{w}}w_{ki}\left(t\right)\right)H\left(\theta_{ki}\left(t\right)\right), \end{array}$$

where the constant *N*_syn_ defines a soft upper bound on the number of active synapses per branch (we used *N*_syn_ = 20 in all simulations), the constant λ > 0 defines how hard this soft bound is enforced, and $\dot{\sigma }$ denotes the derivative of the logistic sigmoid.

The functional term combines a presynaptic activity trace with a postsynaptic term that indicates the presence of a dendritic spike. The exponential presynaptic activity trace with time constant τ_x_ > 0 is given by:

(22) $$\begin{array}{l} x_{i}\left(t\right)=\underset{f\in F_{i}\left(t\right)}{\sum }\exp\left(-\frac{t-t_{\text{pre},i}^{\left(f\right)}}{\tau_{\text{x}}}\right)H\left(t-t_{\text{pre},i}^{\left(f\right)}\right), \end{array}$$

where $F_{i}\left(t\right)=\left\{f:t_{\text{pre},i}^{\left(f\right)}\leq t\right\}$ is the set of all indices of input spikes with spike times before *t*. The presence of a postsynaptic dendritic spike is formalized as a function Γ_*k*_ which is 1 if a plateau potential is present at time *t* and 0 otherwise. More formally, using ${\hat{t}}_{k}\left(t\right)$ to indicate the time of the last branch spike initiation before *t*, i.e., t^k(t)=maxf{tk(f):tk(f)≤t stretchy="false"}, we define

(23) $$\begin{array}{l} \Gamma_{k}\left(t\right)=H\left(t-{\hat{t}}_{k}\left(t\right)\right)-H\left(t-{\hat{t}}_{k}\left(t\right)-\Delta_{k}^{\text{ds}}\left({\hat{t}}_{k}\left(t\right)\right)\right), \end{array}$$

where $\Delta_{k}^{\text{ds}}\left({\hat{t}}_{k}\left(t\right)\right)$ is the length of this plateau potential as defined in Equation (14). The functional term is then given by

(24) $$\begin{array}{l} f_{ki}^{L}\left(t\right)=c_{L}\Gamma_{k}\left(t\right)\left(x_{i}\left(t\right)-\gamma \left(1-x_{i}\left(t\right)\right)\right)H\left(\theta_{ki}\left(t\right)\right) \end{array}$$

with constants $c_{L}>0$ and γ > 0.

#### 4.2.3. STDP

Denoting the postsynaptic somatic spike times by *t*^(1)^, *t*^(2)^, … , we define the postsynaptic spike trains as sums of Dirac delta pulses, i.e., $S\left(t\right)=\underset{f}{\sum }\delta \left(t-t^{\left(f\right)}\right)$. Using this notation, the STDP update term is given by

(25) $$f_{ki}^{STDP}(t)=\{\begin{array}{ll} -c_{STDP}x_{i}(t)S(t), & \text{if }V_{k}^{\text{b}}(t)\geq STDP_{\text{th}}\\ 0, & \text{otherwise}, \end{array}$$

where $c_{\mathrm{STDP}}>0$ is a constant, *x*_*i*_ is an exponential trace of the presynaptic spike times with time constant τ_x_ as defined in Equation (22), and where ${\mathrm{STDP}}_{\text{th}}$ is the threshold for STDP initiation (for parameter values, see Table 2). Note that this update models the induction of depression for a pre-before-post spike pair (an inverted STDP rule) as found for synapses in the distal dendrites (Kampa et al., 2007). The plasticity dynamics with STDP for the parameters θ_*ki*_ is therefore

(26) $$\begin{array}{l} d\theta_{ki}\left(t\right)=\eta H\left(\theta_{ki}\left(t\right)\right)\left(f_{ki}^{S}\left(t\right)+f_{ki}^{L}\left(t\right)+f_{ki}^{STDP}\left(t\right)\right)dt+\sqrt{2\eta T}dW_{ki}. \end{array}$$

Note that we include the term *H*(θ_*ki*_(*t*)) in Equation (26) in order to stress again that only functional synapses are subject to non-stochastic parameter changes.

#### 4.2.4. Alternative Plasticity Rule

To test the dependence of the results on the specific form of the plasticity rule, we considered an alternative rule that models findings in a study by Cichon and Gan (2015). Here, presynaptic activity during a dendritic spike still leads to LTP, but, LTD is induced by presynaptic activity *preceding* a dendritic spike. The functional term in this plasticity rule combines two presynaptic activity traces [with fast (LTP) and slow (LTD) decay] with postsynaptic terms that indicate the onset and the presence of a dendritic spike. The fast decaying presynaptic activity trace $x_{ki}^{\text{LTP}}\left(t\right)$ with time constant $\tau_{{\text{x}}^{\text{LTP}}}=\text{20}\;\text{ms}$ is incremented at each presynaptic spike of neuron *i* that appears within the time period of a dendritic spike of branch *k*. The trace is given by:

(27) $$\begin{array}{l} x_{ki}^{\text{LTP}}\left(t\right)=\underset{f\in F_{ki}^{\text{LTP}}\left(t\right)}{\sum }\exp\left(-\frac{t-t_{\text{pre},i}^{\left(f\right)}}{\tau_{{\text{x}}^{\text{LTP}}}}\right)H\left(t-t_{\text{pre},i}^{\left(f\right)}\right), \end{array}$$

where $F_{ki}^{\text{LTP}}\left(t\right)=\left\{f:t_{\text{pre},i}^{\left(f\right)}\leq t\wedge \Gamma_{k}\left(t_{\text{pre},i}^{\left(f\right)}\right)=1\right\}$ is the set of all indices of input spikes of neuron *i* with spike times before *t* and during the presence of a dendritic spike in branch *k* (recall that the function Γ_*k*_ is 1 during a dendritic spike and 0 otherwise). The slow decaying presynaptic activity trace $x_{ki}^{\text{LTD}}\left(t\right)$ is defined alike. However, this trace is only incremented at each presynaptic spike if there is no postsynaptic dendritic spike. The trace $x_{ki}^{\text{LTD}}\left(t\right)$ with time constant $\tau_{{\text{x}}^{\text{LTD}}}=\text{500}\;\text{ms}$ is defined as

(28) $$\begin{array}{l} x_{ki}^{\text{LTD}}\left(t\right)=\underset{f\in F_{ki}^{\text{LTD}}\left(t\right)}{\sum }\exp\left(-\frac{t-t_{\text{pre},i}^{\left(f\right)}}{\tau_{{\text{x}}^{\text{LTD}}}}\right)H\left(t-t_{\text{pre},i}^{\left(f\right)}\right), \end{array}$$

where $F_{ki}^{\text{LTD}}\left(t\right)=\left\{f:t_{\text{pre},i}^{\left(f\right)}\leq t\wedge \Gamma_{k}\left(t_{\text{pre},i}^{\left(f\right)}\right)=0\right\}$ is the set of all indices of input spikes of neuron *i* with spike times before *t* and in the absence of a postsynaptic dendritic spike. Similar to somatic spike trains, we formalize the dendritic spike train as a sum of Dirac delta pulses at times of dendritic spike onset $\Lambda_{k}\left(t\right)=\underset{f}{\sum }\delta \left(t-t_{k}^{\left(f\right)}\right)$, were $t_{k}^{\left(f\right)}$ denotes the onset time of the *f*^th^ spike of dendrite *k*. The functional term is then given by

(29) $$\begin{array}{l} {\hat{f}}_{ki}^{L}\left(t\right)=\left[c_{L}^{+}x_{ki}^{\text{LTP}}\left(t\right)\Gamma_{k}\left(t\right)-c_{L}^{-}x_{ki}^{\text{LTD}}\left(t\right)\Lambda_{k}\left(t\right)\right]H\left(\theta_{ki}\left(t\right)\right), \end{array}$$

where the constants $c_{L}^{+}=6$ and $c_{L}^{-}=2$ scale the contribution of potentiation and depression. The structural term and the STDP term are not altered and are kept as defined above (Equation (21) and (25), respectively). The alternative plasticity dynamics for the parameters θ_*ki*_ is therefore,

(30) $$\begin{array}{l} d\theta_{ki}\left(t\right)=\eta H\left(\theta_{ki}\left(t\right)\right)\left(f_{ki}^{S}\left(t\right)+{\hat{f}}_{ki}^{L}\left(t\right)+f_{ki}^{STDP}\left(t\right)\right)dt+\sqrt{2\eta T}dW_{ki}. \end{array}$$

### 4.3. Experimental Design and Statistical Analysis

All simulations as well as statistical analyses were performed using Python v3.5.3 with SciPy v0.18.1 and NumPy v1.13.0. The model was simulated with a time step of 1 ms. We used the Euler method for the integration of the deterministic neuron dynamics, and we used the Euler-Maruyama method for the integration of the stochastic dynamics of the synaptic parameters. Every neuron state, that is, membrane potential, remaining refractory period, etc., as well as the synaptic parameters are represented by fixed size state vectors that are update at each time step of the simulation.

D'Agostino-Pearson tests were used to assess normality. Statistical analyses were performed using different tests as appropriate, as stated in the main text and the figure legends. All of the data is presented as mean ± SD. Significant levels for all significance tests were set at *p* ≤ 0.05.

Simulation parameters are listed in Tables 1, 2. All simulations were based on the same parameter sets. Parameters used in the simulations with the alternative plasticity rule are given in section 4.3.3. To investigate the impact of the parameters on the simulation results we performed a one-at-a-time sensitivity analysis (see section 1.2 in the Supplementary Material for more detail).

#### 4.3.1. Details to Simulations for Figure 2

A total of 320 input neurons were divided into eight disjoint assemblies of 40 neurons each. Neurons within each input assembly were preferentially active together. More precisely, at times *t*_start_ + *k*(Δ*t*_act_ + Δ*t*_delay_), with 0 ≤ *k* < *N*_p_ and *N*_p_ being the number of input patterns, one assembly was chosen uniformly at random from the set of all input assemblies and each neuron in the assembly emitted a 35 Hz Poisson spike train (randomly generated at every pattern presentation) for Δ*t*_act_ = 300 ms. In addition, to model background activity, each neuron from the whole input population was spiking at a rate of 1 Hz. The delay between two pattern presentations (the delay between successive active assemblies) was set to Δ*t*_delay_ = 200 ms. During this time and from *t* = 0 to *t*_start_ = 200 ms only the background noise was active. We assume that each input neuron could potentially establish a connection to each branch. Note that this is a reasonable assumption if the neurons are considered to be located within the same cortical column (Chklovskii et al., 2004; Kalisman et al., 2005). Input neurons were initially connected to branches randomly, such that exactly 20 synapses were established on each branch. Synaptic parameters of these connections were independently drawn from a uniform distribution on the interval [4, 8). The soft maximum of synapses per branch (*N*_syn_) was also set to 20. A total of *N*_p_= 2,000 patterns as described above were presented to the neuron. Neuron and plasticity parameters were set according to Tables 1, 2, respectively.

#### 4.3.2. Details to Simulations for Figure 3

Patterns were generated and parameters were set as in simulations for Figure 2. In these simulations, however, we added the STDP update (Equation 25) to the parameter dynamics. All of the following simulations, that is, simulations with alternative plasticity rule, simulations for Figures 4, 5, were conducted with the STDP update.

#### 4.3.3. Details to Simulations With Alternative Plasticity Rule

We repeated the simulations for Figure 3 but with an alternative functional term in the plasticity dynamics (according to Equation 29). We presented a total of *N*_p_= 2,000 patterns to the neuron and we used the following plasticity parameters: $c_{L}^{+}=6$, $c_{L}^{-}=2$, $\tau_{{\text{x}}^{\text{LTP}}}=\text{20}\;\text{ms}$, $\tau_{{\text{x}}^{\text{LTD}}}=\text{500}\;\text{ms}$. All other parameters were set according to to Tables 1, 2. Results of these simulations are given in the Discussion.

#### 4.3.4. Details to Simulations for Figure 4

Input assembly activations and background noise were generated as in the simulations described above (simulations for Figures 2, 3), but instead of choosing one assembly at random at times of pattern presentation, we activated the assemblies sequentially. In other words, we first activated exclusively assembly *A*_1_ 250 times, then assembly *A*_2_, then assembly *A*_3_, etc. The delay between two pattern presentations was set to Δ*t*_delay_ = 200 ms as before. Parameters were set according to Tables 1, 2. At the end of all pattern presentations of an assembly, we evaluated for each branch which, if any, assemblies had synaptic clusters on that branch.

#### 4.3.5. Details to Simulations for Figure 5A

Patterns were generated similar as in simulations for Figure 2, but instead of choosing one assembly at random at times of pattern presentations, up to 4 assemblies were chosen uniformly at random from the set of all input assemblies and were simultaneously activated. In addition, in successive simulations, we reduced the fraction *p* of active neurons in each assembly from 1.0 down to 0.5 (in steps of 0.1). The fraction *p* of assembly neurons was chosen randomly at each pattern presentation. These neurons increased their firing rate to 35 Hz as before, while the rest remained at the background rate of 1 Hz. In each of these simulations we presented a total of *N*_p_ = 2,000 patterns to the neuron. Neuron and plasticity parameters were set according to Tables 1, 2, respectively.

#### 4.3.6. Details to Simulations for Figures 5B,C

These simulations were performed with non-disjoint input assemblies. Non-disjoint assemblies were created by randomly choosing for each assembly either 10, 20, 30, or all 40 neurons from a shared pool of input neurons consisting of 80, 160, 240, and 320 neurons, respectively. Since the number of neurons per assembly and the total number of input neurons were kept as before (40 assembly neurons and 320 input neurons), this procedure led to an overlap between assemblies, where the overlap between a given pair of assemblies was 3.11 ± 2.48% (mean ± SD, over 25 independent trials), 6.15 ± 3.53%, 9.38 ± 4.19%, and 12.5 ± 5.11% for a shared neuron pool of size of 80, 160, 240, and 320, respectively. Patterns of these assemblies were then created as given in details to simulations for Figure 2. A total of *N*_p_ = 2,000 patterns were presented to the neuron. Parameters were set according to Tables 1, 2.

### 4.4. Details to Analysis of Stochastic Rewiring Dynamics

#### 4.4.1. Structural Prior

We first consider the structural plasticity term $f_{ki}^{S}$. Note that this term is meant to model the physical constraint that the number of synapses on a branch is bounded. We used

(31) $$f_{ki}^{S}(t)=\{\begin{array}{ll} -2\lambda c_{\text{w}}c_{\theta }[1-\sigma (\lambda (N_{\text{syn}}-N(\theta_{k}(t))))]\dot{\sigma }(c_{\text{w}}w_{ki}(t)), & \text{if }\theta_{ki}(t)>0\text{ (functional connection)}\\ 0, & \text{if }\theta_{ki}(t)\leq 0\text{ (non-established connection)}, \end{array}$$

where σ denotes the logistic sigmoid function, $\dot{\sigma }$ denotes its derivative, and *N*(**θ**_*k*_) counts the number of active synapses at branch *k* in a smooth manner (see section 4). The constants λ and *c*_w_ will be discussed below. Equation (31) has a very simple interpretation. When the number of synapses at the branch is below *N*_syn_, the term in the brackets is approximately 0, and the structural term has a tiny influence on the dynamics. However, when the number of synapses is above *N*_syn_, this term is approximately 1, which will tend to decrease synaptic efficacies until the desirable synapse population size at the branch is reestablished. Due to the last term, $\dot{\sigma }\left(c_{\text{w}}w_{ki}\left(t\right)\right)$, which is 0.25 for zero weights and then decreases to 0, weak synapses depress faster than stronger ones. This is reasonable since strong synapses are expected to play a more important role in the function of the neuron, and it is consistent with the experimental finding that large dendritic spines tend to be more stable than small ones. The constant *c*_w_ defines how fast this term decreases to 0.

We show below that this structural term in the stochastic dynamics corresponds to a structural prior $p_{S}\left(\theta \right)$ that factorizes into one structural prior per branch

(32) $$\begin{array}{l} p_{S}\left(\theta \right)=\underset{k}{\prod }p_{S}\left(\theta_{k}\right), \end{array}$$

with each distribution given by

(33) $$\begin{array}{l} p_{S}\left(\theta_{k}\right)=\frac{1}{Z_{S}}\sigma \left(\lambda \left(N_{\text{syn}}-N\left(\theta_{k}\right)\right)\right), \end{array}$$

where $Z_{S}$ is a normalizing constant. We can interpret this distribution as follows. At each branch, the synapse count *N*(**θ**_*k*_) on the branch is compared to the maximum number of synapses *N*_syn_. All configurations where the actual number of synapses is below *N*_syn_ have approximately the same probability, while the prior goes to zero quickly for counts above that number. The parameter λ > 0 scales the steepness of the sigmoid and therefore how strictly this bound is enforced. In other words, using this structural prior, the stochastic plasticity dynamics tend to sample network configurations where the number of synapses per branch is at most *N*_syn_.

We now show that the structural plasticity term $f_{ki}^{S}$ follows from the structural prior given in Equations (32), (33). According to Equation (4), we have,

$$\begin{array}{l} f_{ki}^{S}\left(t\right)=\frac{\partial }{\partial \theta_{ki}}\log p_{S}\left(\theta \right){|}_{\theta \left(t\right)}\\ \;=\frac{\partial }{\partial \theta_{ki}}\underset{l}{\sum }\log\left(\frac{1}{Z_{S}}\sigma \left(\lambda \left(N_{\text{syn}}-N\left(\theta_{l}\right)\right)\right)\right){|}_{\theta \left(t\right)}\\ \;=\frac{\partial }{\partial \theta_{ki}}\log\left(\frac{1}{Z_{S}}\sigma \left(\lambda \left(N_{\text{syn}}-N\left(\theta_{k}\right)\right)\right)\right){|}_{\theta \left(t\right)}\\ \;=\frac{\partial }{\partial \theta_{ki}}\log\left(\sigma \left(\lambda \left(N_{\text{syn}}-N\left(\theta_{k}\right)\right)\right)\right){|}_{\theta \left(t\right)} \end{array}$$

(34) $$\begin{array}{l} =-\left(1-\sigma \left(\lambda \left(N_{\text{syn}}-N\left(\theta_{k}\right)\right)\right)\right)\frac{\partial }{\partial \theta_{ki}}N\left(\theta_{k}\right){|}_{\theta \left(t\right)}\\ =-2\lambda c_{\text{w}}c_{\theta }\left[1-\sigma \left(\lambda \left(N_{\text{syn}}-N\left(\theta_{k}\left(t\right)\right)\right)\right)\right]\dot{\sigma }\left(c_{\text{w}}w_{ki}\left(t\right)\right)H\left(\theta_{ki}\left(t\right)\right). \end{array}$$

#### 4.4.2. Functional Likelihood

We next link the functional plasticity term $f_{ki}^{L}$ to the functional likelihood Equations (7), (8). This link is again established using Equation (4). First, we note that

(35) $$\begin{array}{l} \frac{\partial }{\partial \theta_{ki}}\log p_{L}(\theta )=\\ \;{\langle \frac{{\hat{c}}_{L}}{t_{\text{max}}}\int_{0}^{t_{\text{max}}}\frac{\partial }{\partial \theta_{ki}}\Gamma_{k}(s)\left(\sum_{j}w_{kj}x_{j}(s)-\gamma \sum_{j}w_{kj}(1-x_{j}(s))\right)ds\rangle }_{\;X,\Gamma_{k}} \end{array}$$

Under the simplifying assumption that Γ_*k*_(*t*) does not depend on θ_*ki*_ (which is approximately satisfied if each input has only a small influence on the branch potential), we obtain

(36) $$\frac{\partial }{\partial \theta_{ki}}\log p_{L}(\theta )\approx {\langle \frac{{\hat{c}}_{L}c_{\theta }}{t_{\text{max}}}\int_{0}^{t_{\text{max}}}\Gamma_{k}(s)\left(x_{i}(s)-\gamma (1-x_{i}(s))\right)H(\theta_{ki})ds\rangle }_{\;X,\Gamma_{k}}.$$

Assuming slow parameter updates, we approximate this with a self-averaging online update

(37) $$\begin{array}{l} \frac{\partial }{\partial \theta_{ki}}\log p_{L}\left(\theta \right){|}_{\theta \left(t\right)}\approx c_{L}\Gamma_{k}\left(t\right)\left(x_{i}\left(t\right)-\gamma \left(1-x_{i}\left(t\right)\right)\right)H\left(\theta_{ki}\left(t\right)\right). \end{array}$$

## Data Availability Statement

The raw data supporting the conclusions of this article will be made available by the authors, without undue reservation, to any qualified researcher. The Python code that was used to generate the results reported in this article is available at: https://github.com/IGITUGraz/dendritic_rewiring.

## Author Contributions

RL and TL conceived the research idea, wrote the manuscript, analyzed the simulation data, and prepared the figures. TL wrote the code for the computational models and performed the simulations.

## Conflict of Interest

The authors declare that the research was conducted in the absence of any commercial or financial relationships that could be construed as a potential conflict of interest.
