# Supplementary material for: Emergence of Stable Synaptic Clusters on Dendrites Through Synaptic Rewiring
Source: Front Comput Neurosci. 2020 Aug 6;14:57. doi: 10.3389/fncom.2020.00057 (PMC7424032; doi:10.3389/fncom.2020.00057)
Supplement: Supplementary file 1 [file Data_Sheet_1.pdf]

Supplementary Material

1 SUPPLEMENTARY METHODS

1.1 Analysis of the influence of dendritic spikes on synaptic clustering

In our model we considered dendritic branch dynamics including a stochastic firing threshold and dendritic spikes where the shape of the dendritic spike is given by a brief sodium spikelet followed by a plateau. We hypothesized that this nonlinear integration of synaptic input and dendritic plateau potentials are crucial for synaptic clustering in our model. In order to verify this, we conducted simulations of an altered model with linear dendritic integration (i.e., we removed the firing threshold and did not model dendritic spikes). The dynamics of the soma were kept as before.

In the original model, the functional term of the plasticity dynamics was given by

$$f_{ki}^{\mathcal{L}}(t) = \begin{cases} c_{\mathcal{L}}\Gamma_k(t)(x_i(t) - \gamma(1 - x_i(t)), & \text{if } \theta_{ki}(t) > 0 \quad \text{(functional connection)} \\ 0, & \text{if } \theta_{ki}(t) \leq 0 \quad \text{(non-established connection)}, \end{cases} \tag{S1}$$

where $c_{\mathcal{L}} > 0$ and $\gamma > 0$ are constants. The constant γ determines the threshold activity that switches from long-term depression (LTD) to long-term potentiation (LTP) and x_i is the exponential presynaptic activity trace. Note that changes occur only during the presence of a plateau potential (Γ_k is one during a dendritic spike and zero otherwise). In order to adapt these dynamics to the altered model, we introduced a threshold $\mathcal{L}_{\text{th}}$ for LTD/LTP initiation. The adapted functional term of the plasticity dynamics is then given by

$$f_{ki}^{\mathcal{L}}(t) = \begin{cases} c_{\mathcal{L}}\Pi_k(t)(x_i(t) - \gamma(1 - x_i(t)), & \text{if } \theta_{ki}(t) > 0 \quad \text{(functional connection)} \\ 0, & \text{if } \theta_{ki}(t) \leq 0 \quad \text{(non-established connection)}, \end{cases} \tag{S2}$$

where $\Pi_k = 1$ whenever the membrane potential of branch k is above $\mathcal{L}_{\text{th}}$, and $\Pi_k = 0$ if the membrane potential of the respective branch is below this threshold. Hence, the plasticity rule has a similar threshold-like behavior as the original one. The only difference is thus the missing extended depolarization caused by the dendritic plateau potential.

We performed simulations as described for Figure 2 in the main text using the adapted functional term and with various values of $\mathcal{L}_{\text{th}}$ ranging from $-70\,\text{mV}$ to $-55\,\text{mV}$ in $1\,\text{mV}$ steps (with 25 independent trials per value). We could not observe synaptic clustering for any of the considered LTD/LTP thresholds. At low thresholds, a few synapses retracted over time and a few were established. Other synapses showed after an initial increase some fluctuations in their weights (Figure S1). The membrane potentials of the branches exceed these low thresholds for LTD/LTP initiation by the contribution of any input synapse regardless of whether a specific assembly is active or not. The functional term acts on all these synapses which are alternately depressed and potentiated. For medium thresholds, synapses tended to retract over time and weak synapses were established (Figure S2). In this setup $\mathcal{L}_{\text{th}}$ is reached by the contribution of only a few initially strong individual synapses during assembly pattern presentation. These synapses are not able to depolarize the dendritic membrane potential for an extended period of time (as it would be the case with dendritic plateau potentials), hence the contribution of LTP to these synapses is quite weak and synapses that are not active during this short period of time are depressed. For higher thresholds, the initial synapses remained rather stable (Figure S3). For these hight thresholds the membrane potential

of the branches is almost always below $\mathcal{L}_{\rm th}$ and hence there is almost no contribution of the functional term to the synaptic weight. As a consequence the initial synapses remain stable. These results support our initial assumption that nonlinear dendritic integration and dendritic plateau potentials are indeed necessary for assembly-specific clustering of synapses onto individual dendritic branches. In summary, our simulations indicate that the extended depolarization of dendritic plateau potentials is necessary to collectively strengthen correlated inputs which eventually leads to synaptic clustering.

1.2 Sensitivity analysis of parameters

We performed a one-at-a-time sensitivity analysis in order to investigate the impact of all plasticity parameters as well as three neuron parameters on the simulation results. We therefore modified the parameters by $\pm 10\,\%$ (each parameter individually while keeping others at their default values) and analyzed the results of each run using a sensitivity index $\mathrm{SI}\%_i$:

$$\mathrm{SI}\%_i = \left(\frac{100}{p}\right) \cdot \frac{|x_i - x_{\rm ref}|}{x_{\rm ref}}, \tag{S3}$$

where p is the parameter variation in percent ($\pm 10\,\%$), x_i is the number of represented assemblies under perturbation of parameter i averaged over 25 independent trials, and $x_{\rm ref}$ is the number of represented assemblies representing the average over 25 independent simulations with the reference parameter values (i.e., the parameter values reported in the main manuscript). The sensitivity index $\mathrm{SI}\%_i$ gives the variation in the clustering (in $\%$) per percent variation of parameter i. This analysis allowed us to measure the importance of each individual parameter. The higher the $\mathrm{SI}\%_i$ is, the more impact the respective parameter has on the model's performance (measured by the number of represented assemblies on the neuron).

The results of this analysis are given in Figure S4. Note that we varied all parameters by $+10\,\%$ and $-10\,\%$ but we only show the stronger variation for each parameter. The threshold for spike-timing-dependent plasticity (STDP) initiation, that is $STDP_{\rm th}$, has clearly the strongest influence on the clustering. The sensitivity index of $\Delta^{\rm ds}_{\rm max}$ (the maximum duration of a plateau potential) was zero for both considered variations. In order to investigate this further, we reduced $\Delta^{\rm ds}_{\rm max}$ in successive simulations from $300\,\mathrm{ms}$ down to $150\,\mathrm{ms}$ (in steps of $30\,\mathrm{ms}$) and recorded the number of represented assemblies on the neuron. The mean number of represented assemblies deviated from $x_{\rm ref}$ at $\Delta^{\rm ds}_{\rm max} = 150\,\mathrm{ms}$ (although not significantly, $t(24) = 0.47$, $p = 0.64$, unpaired t-test). Reducing the maximum allowed duration of a dendritic spike results in more spikes with shorter duration. These results indicate that this effect has only a minor influence on the clustering. Increasing $\Delta^{\rm ds}_{\rm max}$ had no influence on the number of represented assemblies. Although the sensitivity index is highest for $STDP_{\rm th}$, the influence of this parameter on the clustering is quite low (approximately $1.4\,\%$ variation in the clustering per percent variation of $STDP_{\rm th}$). The influence of all other considered parameters on the clustering is even lower (less than $0.5\,\%$ variation in the clustering per percent variation of these parameters). In summary, this analysis showed that the model is quite robust to parameter variations.

1.3 Analysis of the influence of pattern duration and background interval on clustering

In the simulations so far, we considered a pattern duration of $300\,\mathrm{ms}$ and a background interval of $200\,\mathrm{ms}$, and we presented a total of 2000 patterns in each experiment. Hence, each of the 8 input assemblies was active for $75\,\mathrm{s}$ (on average) throughout a simulation. Here we analyze the influence of the pattern duration and the duration of the background interval, that is the delay between successive patterns, on the model's performance (measured by the number of assemblies represented on the neuron). To analyze this we

performed simulations as described for Figure 3 in the main text but with various combinations of pattern durations and pattern delays (we kept the average time that an assembly was active at $75\,\mathrm{s}$). We found that our rewiring mechanism is, up to some point, quite robust to variations in the pattern duration and the delay between patterns (Figure S5). This holds down to pattern durations of about $100\,\mathrm{ms}$. For shorter pattern durations the performance generally degraded for any of the considered delays between successive pattern presentations. The main problem here is that the duration of a branch spike (up to $300\,\mathrm{ms}$) can span over multiple activations of different assemblies. This leads to the conflation of different assembly activations in the plasticity update of single branches and therefore hinders rewiring. For even shorter pattern durations ($10\,\mathrm{ms}$; data not shown) the rewiring dynamics were not able to separate connections from different assemblies to different branches.

1.4 Analysis of the weakening of older memories over time

In Section 2.4 of the main text we showed that rewiring supports memory protection by recruiting branches sequentially to store input patterns. That is, when activating exclusively an assembly A_1, then an assembly A_2, etc., at first only one or a few branches evolve a synaptic cluster for the first shown assembly while all other branches remain neutral to this assembly. When further assemblies become active, new branches are recruited to store these patterns while synapse cluster at old branches (branches that evolved a synapse cluster to earlier activated assemblies) remain rather stable.

However, due to the random fluctuations of the weights that are imposed by the noise term in our rewiring dynamics, synaptic connections to inactive assemblies gradually degrade. This behavior can be interpreted as gradual forgetting. If an assembly is inactive for some time, synaptic weights of a branch that previously evolved a synaptic cluster to that assembly decrease with time. Ultimately, if an assembly is inactive for an extended period of time, the branch will become neutral to this assembly. The branch can then adapt again to respond to novel input assemblies.

To analyze this weakening of older memories over time we considered a neuron with 12 branches where each of the branches had evolved a synaptic cluster with 20 synapses to one of 8 input assemblies (assemblies A_1 to A_8). Four branches were not specialized to any assembly. We simulated the neuron for $60\,\mathrm{min}$ and every $500\,\mathrm{ms}$ one of 7 assemblies (assemblies A_1 to A_7) was chosen randomly and activated for $300\,\mathrm{ms}$ (assembly patterns and background noise were generated, and parameters were set as described in *Details to simulations for Figure 3* in the main text). Assembly A_8 was never activated during the simulation. We recorded the synaptic weights of the branch that had a synaptic cluster to the inactive assembly (assembly A_8) and analyzed the average weight decrease per time unit. We performed this analysis with three different strengths of the noise term (that is, with three different values of the temperature parameter T). When repeating this experiment in 25 independent trials, the average change of the mean weight of that assembly was $-0.81\,\%/\mathrm{min} \pm 0.06\,\%/\mathrm{min}$ (mean $\pm$ standard deviation), $-1.08\,\%/\mathrm{min} \pm 0.07\,\%/\mathrm{min}$, and $-1.23\,\%/\mathrm{min} \pm 0.07\,\%/\mathrm{min}$ for temperature values of 0.1, 0.3, and 0.5, respectively (Figure S6A, blue bars; the changes given are relative to the mean of the initial weights). Figure S6B shows the mean weight (blue solid line) of that assembly as a function of time for $T = 0.3$ (the value of the temperature parameter that was used in all simulations reported in the main text). The mean weight does not decrease linearly with time. The mean weight decreased after $30\,\mathrm{min}$ by $\approx 40\,\%$ of its initial value and by $\approx 60\,\%$ after $60\,\mathrm{min}$ of simulated time.

1.5 Retaining older memories for an extended period of time

The deterministic part of our rewiring dynamics contains a structural plasticity term $f_{ki}^{\mathcal{S}}$ that follows from a structural prior $p_{\mathcal{S}}$. This structural term is meant to limit the number of functional synapses per branch to N_{syn} in a smooth manner (see Section 2.6 in the main text). We show here that the lifetime of older memories can be increased by using an additional Gaussian prior $p_{\mathcal{G}}(\theta_{ki})$ per synapse ki given by

$$p_{\mathcal{G}}(\theta_{ki}) = \frac{1}{\sigma_{\mathcal{G}}\sqrt{2\pi}} e^{-\frac{1}{2}\left(\frac{\theta_{ki}-\mu_{\mathcal{G}}}{\sigma_{\mathcal{G}}}\right)^2} \tag{S4}$$

with mean $\mu_{\mathcal{G}}$ and standard deviation $\sigma_{\mathcal{G}}$. By using this Gaussian prior $p_{\mathcal{G}}$ in combination with the structural prior $p_{\mathcal{S}}$ the stochastic plasticity dynamics tend to sample network configurations where the weight of a functional synapse is close to $\mu_{\mathcal{G}}$ and where the number of these functional synapses per branch is at most N_{syn}. The Gaussian prior will mitigate the influence of the noise term and thus helps to stabilize the weights of a synapse cluster of a branch even in the absence of input activity. To quantify this, we have repeated the experiment as described in Section 1.4 (above) with stochastic dynamics of parameters θ_{ki} given by

$$d\theta_{ki}(t) = \eta H(\theta_{ki}(t))\Big(f_{ki}^{\mathcal{S}}(t) + f_{ki}^{\mathcal{G}}(t) + f_{ki}^{\mathcal{L}}(t) + f_{ki}^{\mathcal{STDP}}(t)\Big)dt + \sqrt{2\eta T}d\mathcal{W}_{ki}, \tag{S5}$$

where the new term $f_{ki}^{\mathcal{G}}(t) = -\frac{\theta_{ki}(t)-\mu_{\mathcal{G}}}{\sigma_{\mathcal{G}}^2}$ follows from the Gaussian prior. The mean $\mu_{\mathcal{G}}$ was set to 8, that is the value of $w_{\max}$, and the standard deviation $\sigma_{\mathcal{G}}$ was set to 32. In 25 independent simulations of this experiment, the average change of the mean weight of an assembly that hosted a synaptic cluster from an inactive assembly changed from $-0.81\,\%/\mathrm{min}$ to $-0.25\,\%/\mathrm{min} \pm 0.02\,\%/\mathrm{min}$ (mean $\pm$ standard deviation), from $-1.08\,\%/\mathrm{min}$ to $-0.33\,\%/\mathrm{min}\pm 0.05\,\%/\mathrm{min}$, and from $-1.23\,\%/\mathrm{min}$ to $-0.48\,\%/\mathrm{min}\pm 0.06\,\%/\mathrm{min}$ for temperature values of 0.1, 0.3, and 0.5, respectively (Figure S6A, red bars; the changes given are relative to the mean of the initial weights). Again, the mean weight does not decrease linearly with time (Figure S6B, red dotted line). After an initial decrease to $\approx 80\,\%$ of its initial value it remains rather stable.

We next asked whether this additional Gaussian prior influences the model's performance (measured by the number of represented assemblies on the neuron). To test this, we performed simulations as described for Figure 4 in the main text using the additional term $f_{ki}^{\mathcal{G}}$. We found that the average number of represented assemblies increased from 6.92 ± 0.89 to 7.64 ± 0.48 (mean $\pm$ standard deviation over 25 independent trials). This is a significant increase of the number of assemblies that are stored on the dendrites ($t(24) = 3.49$, $p = 0.001$, unpaired t-test). In summary, using an additional Gaussian prior on the parameters does not only protect memories over longer time scales but also increases the number of patterns that are stored on the neuron.

1.6 Synaptic consolidation by sharpening of weight priors

In Section 1.5 (above) we described a way to reduce gradual forgetting in our model by introducing an additional term $f_{ki}^{\mathcal{G}}$ in the rewiring dynamics. This term resulted from a Gaussian prior $p_{\mathcal{G}}$ on the parameters θ_{ki}. By using this additional Gaussian prior the stochastic plasticity dynamics tend to sample network configurations where the weight of a functional synapse is close to the mean of $p_{\mathcal{G}}$. Note that, due to the functional term $f_{ki}^{\mathcal{S}}$, the number of synapse per branch is still bounded (we used a soft upper bound of $N_{\mathrm{syn}} = 20$ per branch in all simulations).

Here, we investigate a simple way how consolidation can be incorporated in this framework by sharpening of the Gaussian prior. Note that, the sharper this prior, the more probability mass is concentrated directly around its preferred synaptic weight. Hence, weights will tend to stay in the vicinity of the mean of the prior (see Section 2.6 in the main text for a description of the parameter sampling dynamics of the network). In this consolidation mechanism, the standard deviation of the Gaussian prior is a function of the synaptic parameter θ_{ki}. Consolidation of a synapse occurs whenever its weight reaches the threshold for synaptic consolidation. Consolidated synapses are then well protected against changes induced by the noise term. More precisely, the standard deviation $\sigma_{\mathcal{G}}$ is given by

$$\sigma_{\mathcal{G}}(\theta_{ki}) = \begin{cases} \sigma_{\mathcal{G}}^{\mathrm{h}}, & \text{if } \theta_{ki}(t) \geq \mathcal{G}_{\mathrm{th}} \\ \sigma_{\mathcal{G}}^{\mathrm{l}}, & \text{otherwise,} \end{cases} \tag{S6}$$

where $\sigma_{\mathcal{G}}^{\mathrm{h}} = 1$, $\sigma_{\mathcal{G}}^{\mathrm{l}} = 32$, and where $\mathcal{G}_{\mathrm{th}} = 7$ is the threshold for synaptic consolidation (note that we apply the Gaussian prior only to functional synapses, see Equation S5). We hypothesized that a branch that hosts a synaptic cluster from one of the input assemblies, receiving approximately 20 synapses from that assembly with the weight at or above $\mathcal{G}_{\mathrm{th}}$, will be strongly protected in this way. To quantify this, we have repeated the experiment as described in Section 1.4 (above) where we added this consolidation mechanism. In 25 independent simulations of this experiment, the average change of the mean weight of an assembly that hosted a synaptic cluster from an inactive assembly was $-0.002\,\%/\mathrm{min} \pm 0.0004\,\%/\mathrm{min}$ (mean $\pm$ standard deviation), $-0.003\,\%/\mathrm{min} \pm 0.0006\,\%/\mathrm{min}$, and $-0.003\,\%/\mathrm{min} \pm 0.0005\,\%/\mathrm{min}$ for temperature values of 0.1, 0.3, and 0.5, respectively (Figure S6A, green bars; the changes given are relative to the mean of the initial weights). The mean weight is stabilized and does virtually not change anymore (Figure S6B, green dashed dotted line).

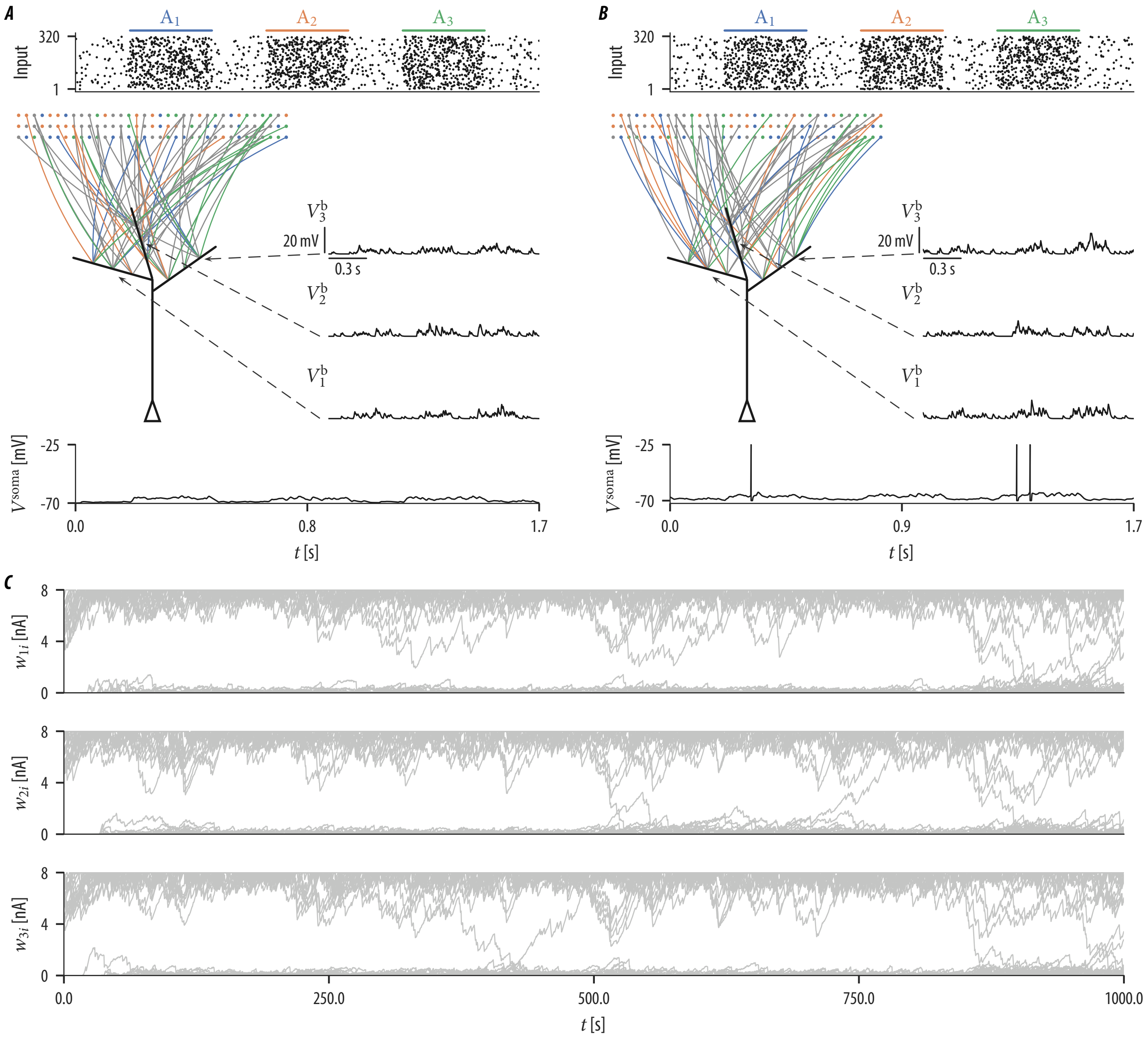

Figure S1. Results of the model with linear dendritic integration where the threshold $\mathcal{L}_{\rm th}$ for LTD/LTP initiation was set to $-69\,\rm mV$. **(A)** Spike raster plot of input neurons for three test patterns of input assemblies $\rm A_1$, $\rm A_2$, and $\rm A_3$ (top; black dots denote spike times), initial wiring diagram of three selected branches ($\rm b_1$, $\rm b_2$, and $\rm b_3$; middle left), dendritic membrane potential $V_i^{\rm b}$ of these branches (middle right), and the somatic membrane potential $V^{\rm soma}$ (bottom). The color of the graph edges indicate to which of the shown assemblies a connection was established (connections in gray are connections to one of the other 5 assemblies). Initially, input neurons were connected to branches randomly such that exactly 20 synapses were established on each branch. The dendritic membrane potential increased during presynaptic assembly activation but this increase was not specific to any of the input assemblies. The somatic membrane potential was subthreshold during pattern presentation of these input assemblies. **(B)** Same as **(A)** but after $17\,\rm min$ of rewiring dynamics. Synapses did not cluster in an assembly-specific way and the dendritic membrane potential was not specific to presynaptic assembly activations. **(C)** Evolution of the synaptic weights w_{ki} of branch $\rm b_1$ (top), $\rm b_2$ (middle), and $\rm b_3$ (bottom). A few synapses retract over time, a few strong, and some weak synapses are established.

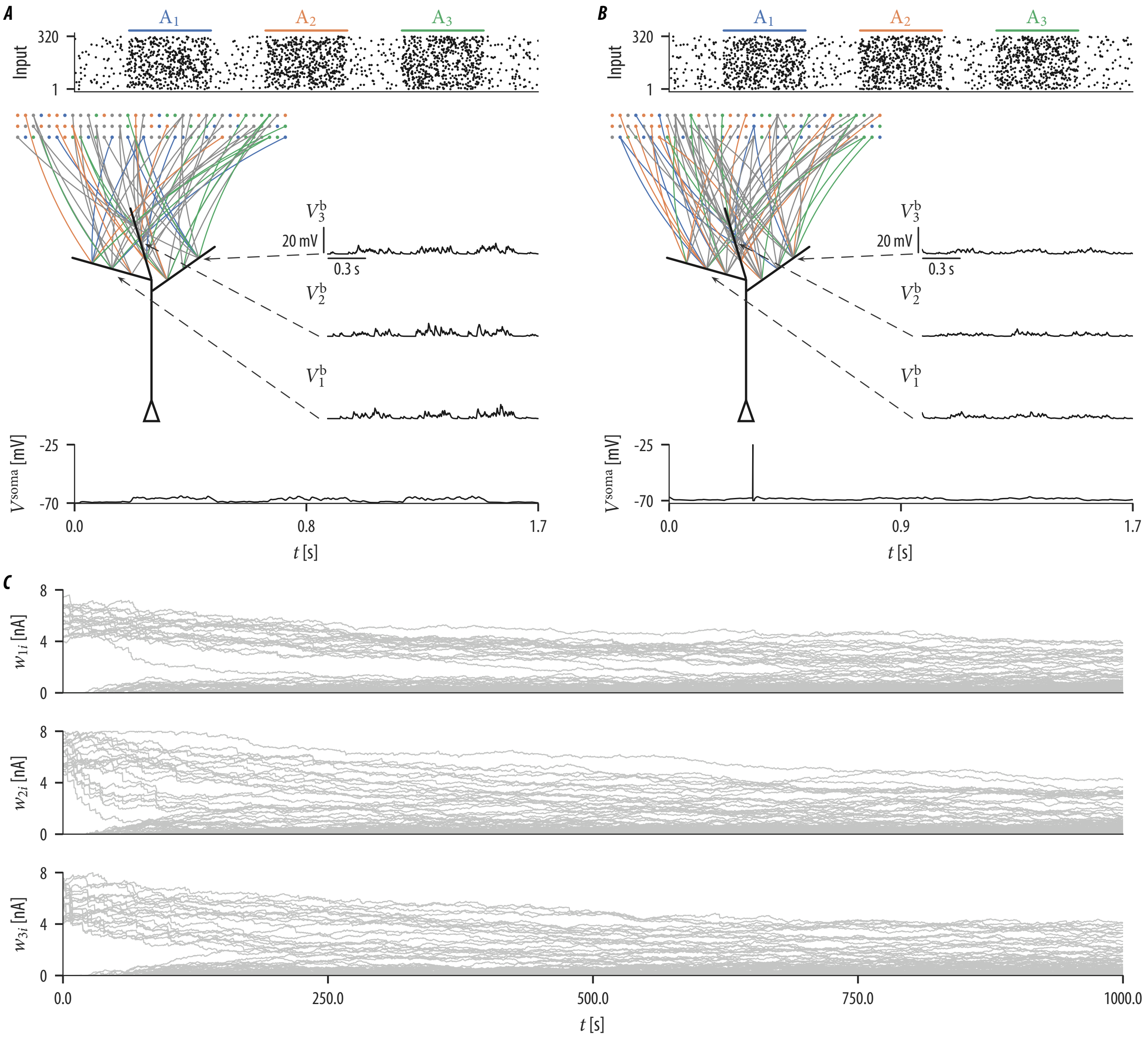

Figure S2. Same as Figure S1 but with LTD/LTP initiation threshold set to $-65\,\mathrm{mV}$. **(A)** Spike raster plot of input neurons (top), wiring diagram of three selected branches (middle left), dendritic and somatic membrane potentials before learning (middle right and bottom) and **(B)** after learning. Evolution of the synaptic weights w_{ki} of branch b_1 (top), b_2 (middle), and b_3 (bottom). Synapses retract over time and weak synapses are established.

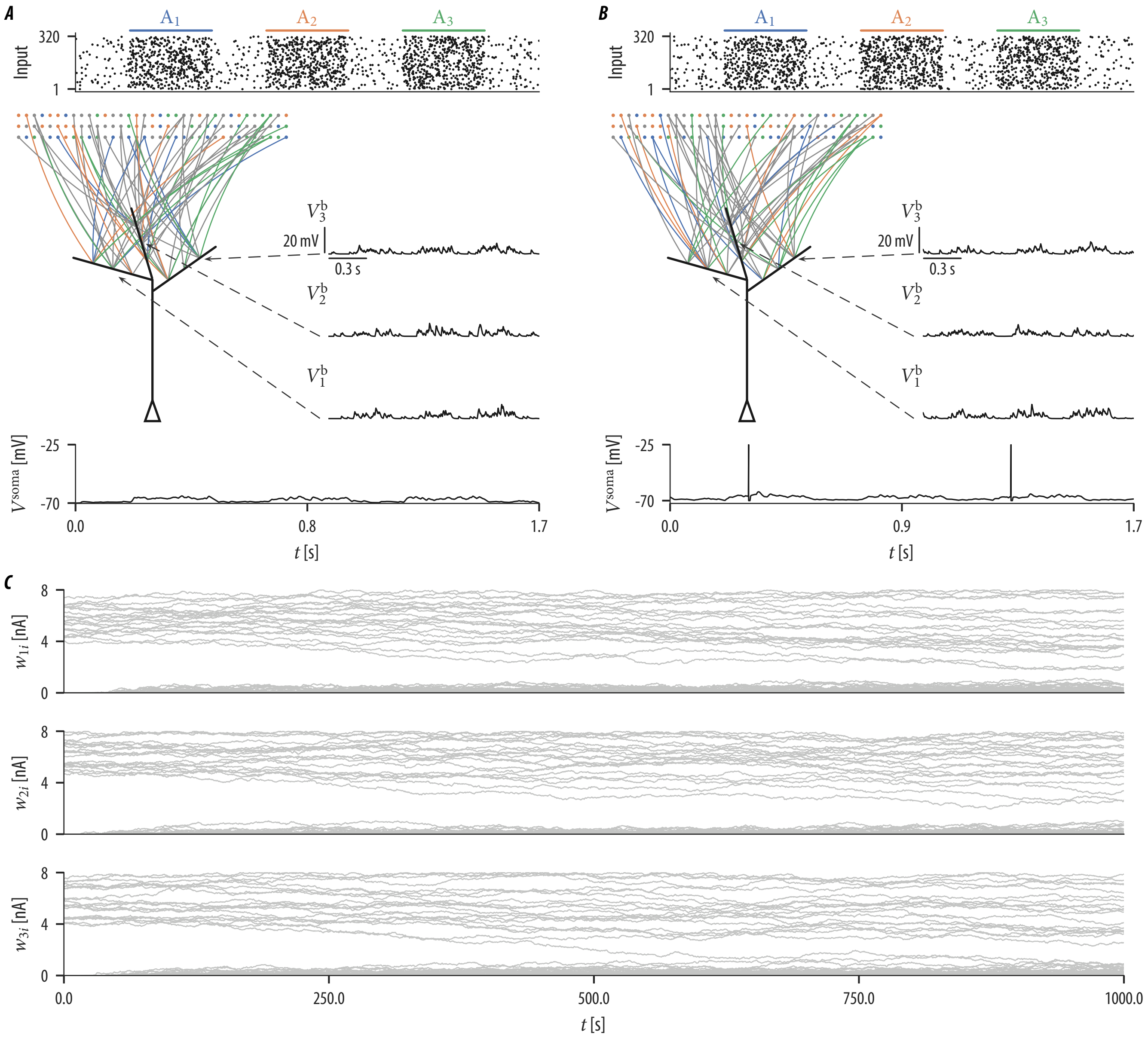

Figure S3. Same as Figure S1 but with LTD/LTP initiation threshold set to $-55\,\mathrm{mV}$. **(A)** Spike raster plot of input neurons (top), wiring diagram of three selected branches (middle left), dendritic and somatic membrane potentials before learning (middle right and bottom) and **(B)** after learning. Evolution of the synaptic weights w_{ki} of branch b_1 (top), b_2 (middle), and b_3 (bottom). Initial synapses remain rather stable and some weak synapses are established.

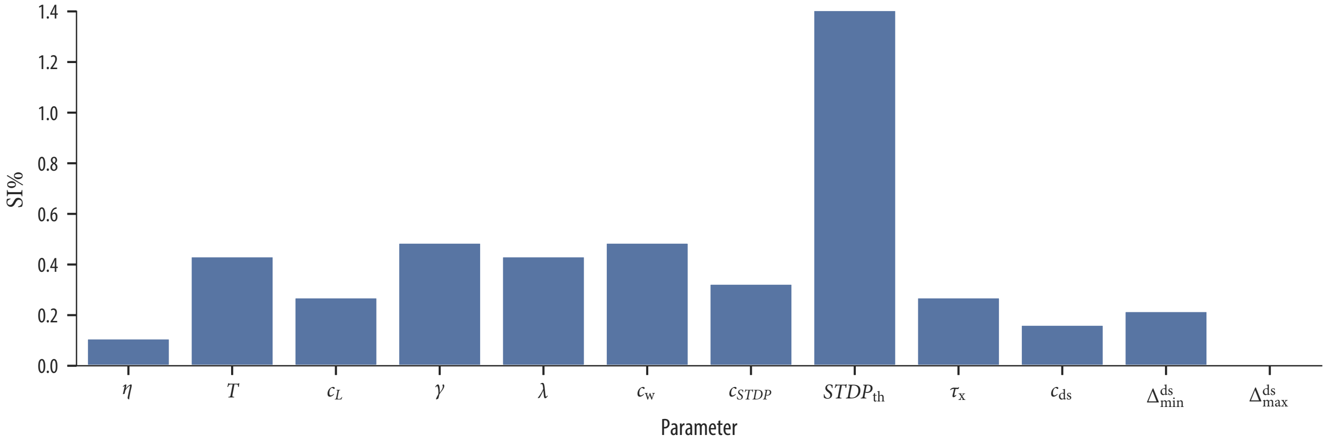

Figure S4. Results of the one-at-a-time sensitivity analysis. Parameters were varied by $\pm 10\,\%$ and the sensitivity index $\mathrm{SI}\%$ for the variation which had the stronger influence on the model's performance is shown. For a description of the parameters see Tables 1 and 2 in the main text.

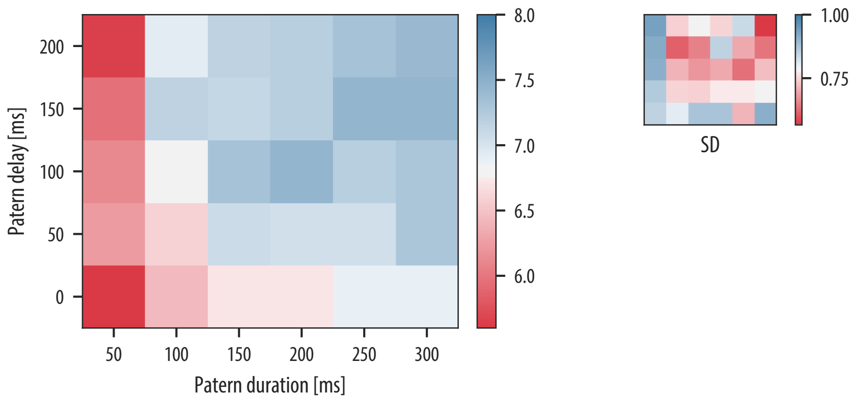

Figure S5. Number of represented assemblies on the neuron as a function of the pattern duration and the delay between patterns. Shown is the mean and standard deviation (inset) over 25 independent trials. Our rewiring mechanism is quite insensitive to variations in the pattern duration and the delay between successive pattern presentations. Short pattern durations combined with short delays had the most adverse impact on the model's performance. The performance generally degraded for pattern durations of $50\,\mathrm{ms}$ (for any of the considered delays).

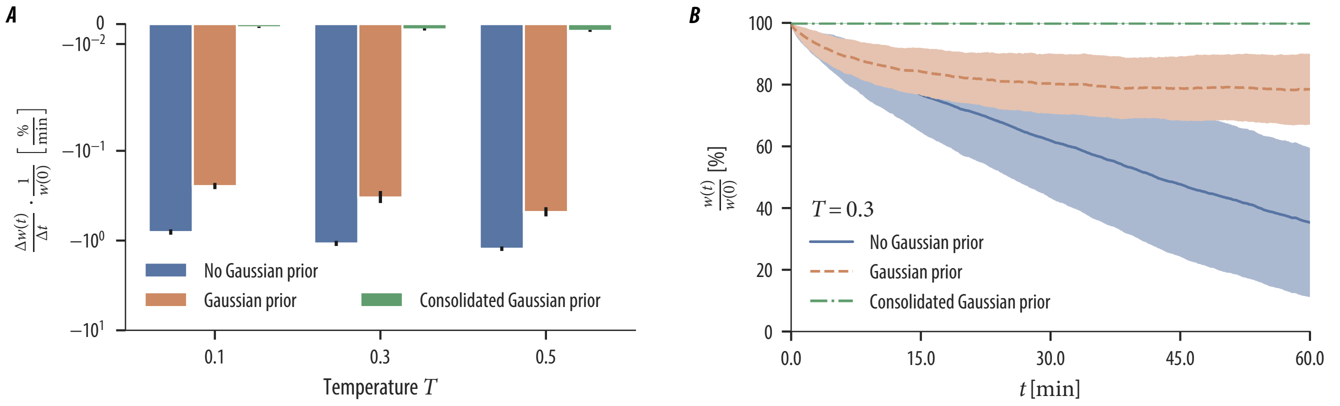

Figure S6. Weakening of older memories over time due to the noise term. **(A)** Average change of the mean weight per time unit of a branch that hosted a synaptic cluster from an inactive assembly for three values of the temperature parameter T. Shown is the mean and the standard deviation over 25 independent simulations of the original model without Gaussian prior (blue), the model with Gaussian prior (red), and the model with consolidated Gaussian prior (green). The values are given relative to the mean of the initial weights (note the logarithmic scale of the y-axis). **(B)** Mean and standard deviation (shaded area) of the weights of an assembly that hosted a synaptic cluster from an inactive assembly as a function of time t (temperature $T = 0.3$; mean and standard deviation is over 25 independent trials and values shown are relative to the initial weights $w(0)$). Results are shown for the model without Gaussian prior (blue solid line), the model with Gaussian prior (red dashed line), and the model with consolidated Gaussian prior (green dashed dotted line). Synapses in our model with consolidated Gaussian prior are well protected against changes induced by the noise term.
